# The Attentional Drift Diffusion Model of Simple Perceptual Decision-Making

**DOI:** 10.3389/fnins.2017.00468

**Published:** 2017-08-24

**Authors:** Gabriela Tavares, Pietro Perona, Antonio Rangel

**Affiliations:** ^1^Computation and Neural Systems, California Institute of Technology Pasadena, CA, United States; ^2^Division of Humanities and Social Sciences, California Institute of Technology Pasadena, CA, United States

**Keywords:** attention, drift-diffusion model, perception, decision-making, eye-tracking

## Abstract

Perceptual decisions requiring the comparison of spatially distributed stimuli that are fixated sequentially might be influenced by fluctuations in visual attention. We used two psychophysical tasks with human subjects to investigate the extent to which visual attention influences simple perceptual choices, and to test the extent to which the attentional Drift Diffusion Model (aDDM) provides a good computational description of how attention affects the underlying decision processes. We find evidence for sizable attentional choice biases and that the aDDM provides a reasonable quantitative description of the relationship between fluctuations in visual attention, choices and reaction times. We also find that exogenous manipulations of attention induce choice biases consistent with the predictions of the model.

## Introduction

Over the last two decades, neuroscientists and psychologists have devoted considerable effort to understanding the neurocomputational basis of decision-making. The goal has been to understand which are the variables encoded at the time of decision, what are the algorithms used to combine them into a decision, and how are these processes implemented and constrained by the underlying neurobiology. Considerable progress has been made in understanding simple perceptual decisions (e.g., determine the net direction of motion in a field of noisy moving dots) and simple value-based choices (e.g., choose between two food snacks). Interestingly, a qualitatively similar class of algorithms has been shown to provide a good description for the accuracy and reaction time patterns in both perceptual (Ratcliff and Rouder, 1998; Gold and Shadlen, 2001, 2007; Smith and Ratcliff, 2004; Ditterich, 2006; Brunton et al., 2013) and value-based choices (Mormann et al., 2010; Tsetsos et al., 2011; Hunt et al., 2012; Philiastides and Ratcliff, 2013; Hutcherson et al., 2015), although many important details remain to be worked out (Bogacz, 2007; Summerfield and Tsetsos, 2012; Tsetsos et al., 2012b; Brunton et al., 2013; Orquin and Loose, 2013; Shadlen and Kiani, 2013; Teodorescu and Usher, 2013). Despite important differences among the various models that have been proposed, all of the algorithms are built around the idea that decisions are made by accumulating noisy evidence in favor of the different alternatives, and that choices are made when the weight of accumulated evidence in favor of one of the options becomes sufficiently strong. For this reason, they are often described as sequential integration models. There is also a growing understanding of how the brain implements these processes in both perceptual (Shadlen and Newsome, 2001; Roitman and Shadlen, 2002; Heekeren et al., 2004; Philiastides et al., 2006; Churchland et al., 2008; Kiani et al., 2008; Tosoni et al., 2008; Ho et al., 2009; Bennur and Gold, 2011; O'Connell et al., 2012) and value-based choice (Basten et al., 2010; Philiastides et al., 2010; Hare et al., 2011b; Hunt et al., 2012; Polanía et al., 2014; Rustichini and Padoa-Schioppa, 2015).

Since many decision tasks require the comparison of spatially distributed stimuli, two important questions are whether decisions are affected by how visual attention is deployed during the process of choice, and if so, how do sequential integrator models need to be modified to incorporate the role of attention. For example, in the context of perceptual choice, if a subject is shown two lines of different length on the left and right of the screen and has to decide which one is longer, how does the pattern of fixations to the two stimuli affect his decision, if at all? Or in the context of value-based choice, if a subject is shown two food stimuli, how does his pattern of fixations affect which of the two he chooses to eat?

This problem has been studied in the realm of value-based choice. Krajbich et al. (Krajbich et al., 2010, 2012; Krajbich and Rangel, 2011; Towal et al., 2013) found that a modification of the popular Drift Diffusion Model, which they call the attentional Drift Diffusion Model (aDDM), provides a quantitatively accurate description of the relationship between visual attention, choices and reaction times in several value-based tasks. The aDDM builds on previous work by Busemeyer and collaborators, who proposed an alternative class of sequential integrator models in which attention plays a role (Roe et al., 2001). In the aDDM, attention influences choices by increasing the relative weight given to evidence related to the attended stimulus. As a result, the model predicts that exogenous shifts of attention can cause systematic choice biases, which is consistent with the results of several studies (Shimojo et al., 2003; Armel et al., 2008; Hare et al., 2011a; Pärnamets et al., 2015; Kunar et al., 2017).

Given that a remarkably similar set of algorithms have been shown to be at work in perceptual and value-based choice tasks in which attention plays no role, it is natural to hypothesize that the aDDM might also provide a reasonable computational description of the role of visual attention in simple perceptual decisions, and that exogenous shifts in attention (i.e., unrelated to the perceptual properties of the stimuli) might causally bias choices as predicted by the aDDM. Here we present the results of two experiments designed to test these hypotheses.

Testing the extent to which the aDDM is able to provide a satisfactory quantitative description of the role of visual attention in simple perceptual choices is interesting for several reasons. First, previous experiments have shown that attention can affect perceptual choices using divided attention paradigms (Wyart et al., 2015), spatial pre-cuing paradigms (Posner et al., 1980; Smith et al., 2004; Carrasco, 2011), and serial dependence paradigms (Fischer and Whitney, 2014). However, the algorithmic or computational description of this effect remains an open question (Summerfield et al., 2013). Second, an important open question in cognitive neuroscience is whether the same algorithms are at work in different domains and systems whenever the problem they are trying to solve is sufficiently similar. This view is consistent with the fact that sequential integrator models are able to accurately describe two-alternative forced choices in domains ranging from memory, to perception, to economic choice (Gold and Shadlen, 2007; Ratcliff and Mckoon, 2008; Starns et al., 2012; Shadlen and Kiani, 2013). However, since perception and value-based choice are made on the basis of different evidence (i.e., perceptual inputs vs. reward predictions), attention might operate through very different channels in these two cases, and thus we cannot assume ex-ante that it might have a computationally similar effect in both types of decisions.

## Materials and methods

### Subjects

In Experiment 1 we tested 25 subjects (10 female, mean age 23), which included Caltech students and staff as well as members of the surrounding community. Subjects were advised to use glasses for eyesight correction as needed. Each subject completed 1,344 decision trials, split into 4 identical experimental sessions, spread across 4 different days. Subjects received a $15 show-up fee in each day, a $40 bonus for completing all sessions, as well as additional earnings based on performance, as described below. In Experiment 2 we tested 20 subjects (9 female, mean age 25). Each subject completed 336 trials in a single session, and received a $15 show-up fee, as well as additional earnings based on performance. The experiments were approved by Caltech's IRB and all subjects provided informed consent prior to participation.

### Experiment 1

**Figure 1:**
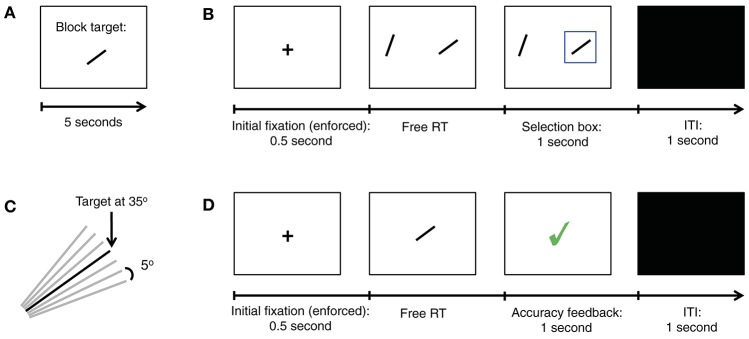
Summary of Experiment 1. **(A)** In the beginning of each block of trials a new target orientation was shown for 5 s. The target was shown again immediately after the training trials, and again after every 5 decision trials. **(B)** Trial structure for our simple perceptual decision task. In each trial subjects must choose the stimulus (left or right) with the orientation closest to the target. **(C)** Diagram showing all seven possible item orientations, in increments of 5°, given a target oriented at 35°. **(D)** Trial structure for training trials.

Experiment 1 consisted of four identical sessions, collected on four separate days, within a period of 2 weeks. Each experimental session was divided into 12 blocks of 28 decision trials. At the beginning of each block, subjects were shown for 5 s a line depicting a target orientation chosen from the set {20°, 35°, 55°, 70°}, as shown in Figure 1A. This excludes vertical and horizontal orientations, which would have made subsequent choices too easy. Each orientation was chosen as the target three times per session, in random order.

In each decision trial subjects were shown two oriented lines, on the left and right sides, with an eccentricity of 16 degrees from the center of the screen (Figure 1B). The relative orientation between each of the two lines and the target, denoted by Δ, was chosen from the set {−15°, −10°, −5°, 0°, 5°, 10°, 15°} (Figure 1C). The subjects' task was to decide which of the two lines had an orientation closest to the target. They were allowed to take as long as needed to make a choice, and indicated their choice with a button press (“A” for left and “L” for right). Let Δ_*left*_ and Δ_*right*_ denote the relative angular distance between the target and the left and right items, respectively. The two choice stimuli shown in the trial were not allowed to have the same Δ. Uniform sampling subject to these constraints led to 42 different trial conditions. Each was used eight times per session, in random order. Subjects saw a blue box around the chosen item in each trial, but they did not receive feedback about the correctness of their decisions during the task.

Stimuli were presented on a 1,280 × 1,024 screen, placed ~50 cm from the subjects' eyes. Subjects were required to keep their hands on the response buttons for the entire task, so they could enter responses without looking at the keyboard. Subjects' fixation patterns were recorded at 500 Hz using an EyeLink 1000 Plus desktop-mounted eye-tracker with head support. Fixations and saccades were determined using the eye-tracker's accompanying software package. The eye-tracking system was calibrated at the beginning of each session, and again whenever the eye-tracker lost the subject's eye (which only occurred 4 times during all sessions of both experiments). Before each decision trial, subjects were required to maintain a continuous fixation on a central cross for 500 ms before the items would appear, which ensured that every trial began with a fixation on the same central location.

In order to familiarize subjects with the targets, they also completed a training task at the beginning of each block (Figure 1D). Here, subjects were shown a single oriented line in the center of the screen, and had to decide whether or not the line shown had the same orientation as the target. They were allowed to take as long as needed to make the decision, which they then indicated with a button press (“A” for no and “L” for yes). Subjects received immediate feedback for 1 s after every decision indicating its correctness. The training task ended after six correct decisions in a row.

The target stimulus for each block was shown once at the beginning of the block (before the training trials), once immediately after the training trials, and again after every 5 decision trials. At the end of each experiment session, we selected 25 decision trials at random, and subjects received an additional payment of $1 for each correct response.

### Experiment 2

**Figure 2:**
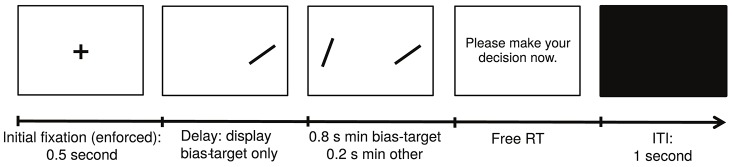
Summary of Experiment 2.

The structure of a typical trial in Experiment 2 is depicted in Figure 2. Experiment 2 was similar to Experiment 1, except for the following differences. First, each subject completed a single session with 12 blocks of 28 trials each. Second, in each trial we randomly selected one of the two items on the screen to be the bias-target item. We used the following procedure to bias fixations toward that item. Unbeknownst to the subjects, we required a minimum amount of cumulative fixation time to each item: 800 ms for the bias-target item and 200 ms for the other one. In every trial we kept track of the cumulative fixation durations to each stimulus and, as soon as the minimum requirement for *both* was met, the items disappeared and the subject was prompted to make a choice. Third, subjects were told (without deception) that both the duration of decision trials and the item that appeared first would be chosen at random every trial, but were not told that the procedure was designed to bias fixations. To minimize awareness of the nature of the experimental manipulation, which relies on giving subjects control over the duration of trials through their fixations, we set the maximum duration for each evaluation period to 3 s. If the minimum fixation requirements for both items were not met within that period of time, the subject was prompted to make a decision. Trials in which the 3-s boundary was binding were removed from additional analyses since they exhibited unusual fixation patterns (24.3% over the entire group; across subjects, min = 6.8% and max = 55.6%). This trial exclusion rule was chosen a priori to minimize the chance that subjects would become aware of the experimental manipulation. Furthermore, the exclusion of these trials did not qualitatively affect any of the reported results. Fourth, subjects were only allowed to enter their decisions after the decision prompt appeared, and could take as much time as needed to do so. Fifth, we refer to trials in which the bias-target item was fixated longer (and in which the stopping condition was reached before 3 s) as *effective* manipulation trials. Note that not all trials were effective since the contingencies described above allow for the possibility that subjects fixate more on the non-bias-target item. In order to increase the fraction of effective trials, which is the manipulation of interest, the bias-target item was always displayed on the screen first, and the other item was only added after a certain delay, which counted toward the total fixation time for the bias-target. The duration of this lag was between 100 and 500 ms, and was calibrated separately for each subject at the beginning of the experiment using the following staircase procedure. The lag started at a value of 300 ms, and was adjusted with a step of 30 ms. After every 3 consecutive effective trials (i.e., trials in which the bias-target item was fixated longer) the lag was decreased by 30 ms, and after a single ineffective trial (i.e., one where the bias-target item was not fixated longer), the lag was increased by the same amount. A total of 48 trials were used in the staircase procedure, and the value of the delay at the 48th trial was then used throughout the remainder of the task (duration: mean = 450 ms, *SD* = 35 ms). Sixth, at the end of the experiment we randomly selected 20 decision trials, and subjects received an additional payment of $1 for each correct response in this set. Seventh, at the end of the task, subjects completed a questionnaire in which we asked if they found anything strange about the timing of the items being displayed and the decision prompt. None of the subjects reported finding a connection between their fixations and the duration of the trials.

### Fixations

All recorded fixations were classified as either item fixations (to either the left or right stimuli on the screen) or “blank” fixations. Trials in which “blank” fixations accounted for more than 50% of the response time were discarded from further analysis (mean percentage discarded trials across all subjects: 5.2%, min = 0.15%, max = 25.8%). Furthermore, if a “blank” fixation was recorded between two fixations to the same stimulus, the observation was converted into a fixation on that item. This is justified by the fact that this type of “blank” fixations tend to be very short, and are likely to be the result of blinking or eye-tracker noise (duration: mean = 41 ms, *SD* = 133 ms). If a “blank” fixation was recorded between fixations on different items, then it was grouped into that trial's inter-fixation transition time and used as such in the analyses below.

### Group model fitting

We used maximum likelihood estimation (MLE) to fit the aDDM to the pooled group data. The model has three free parameters (*d*, θ, and σ) which we fitted using only the odd-numbered trials, so that the even-numbered trials could be used to test its out-of-sample predictions (see the Results section for a description of the aDDM).

The MLE procedure was carried out in multiple steps. In step 1 we defined a coarse grid of parameter combinations, denoted by Ω_1_, which was given by the cross product of the sets {0.001, 0.005, 0.01} for *d*, {0.1, 0.5, 0.9} for θ, and {0.01, 0.05, 0.1} for σ. Although this set only has nine points, it was selected because it spans a wide range of potential parameter combinations. We computed the likelihood of the choice and RTs observed in the odd-numbered trials, conditional on the observed pattern of fixations in each trial, for each vector of parameters in Ω_1_. This was done by simulating the aDMM using the algorithm described in the Supplementary Materials. All time data (including RTs, latencies, fixation durations and inter-fixation transition times) were binned into 10 ms steps. We then selected the vector of parameters in Ω_1_ with the highest log-likelihood as the first candidate solution, which for our data was given by *d* = 0.005, θ = 0.1 and σ = 0.05. Let (*d*_1_, θ_1_, σ_1_) and *ML*_1_ denote, respectively, the best set of parameters and the likelihood that it explains the data that arises from the first step.

The algorithm then proceeded inductively until a stopping criterion was reached. Let Ω_*t*_ denote the search set used in step *t*, and (*d*_*t*_, θ_*t*_, σ_*t*_) and *ML*_*t*_ denote the best candidate solution at this step. Step *t* + 1 then proceeded as follows. A new grid of 9 potential vector parameters, denoted by Ω_*t* + 1_, was constructed as the cross product of the sets {$d_{t}-\frac{\Delta d_{t}}{2}$, *d*_*t*_, $d_{t}+\frac{\Delta d_{t}}{2}$}, {$\theta_{t}-\frac{\Delta \theta_{t}}{2}$, θ_*t*_, $\theta_{t}+\frac{\Delta \theta_{t}}{2}$}, and {$\sigma_{\;t}-\frac{\Delta \sigma_{\;t}}{2}$, σ_*t*_, $\;\sigma_{\;t}+\frac{\Delta \sigma_{\;t}}{2}$}, where Δ*d*_*t*_, Δθ_*t*_ and Δσ_*t*_ correspond to the parameter step sizes used in Ω_*t*_. Note that Ω_*t* + 1_ included (*d*_*t*_, θ_*t*_, σ_*t*_), as well as a finer grid around it.

The MLE step was then repeated again. The algorithm continued until the improvement in the MLE of the proposed parameter solution was <1%. For our data, the convergence process was accomplished in 7 steps, and resulted in an estimate of *d* = 0.0041, σ = 0.063, and θ = 0.36.

### Out-of-sample group simulations

In order to test the ability of the model to predict out of sample, we used the aDDM with the best fitting parameters for the odd-numbered trials to predict data group patterns in the even-numbered trials.

Critically, the predictions were made conditional on the relative orientation of the stimuli, but not on the actual fixation patterns observed in the even trials. To understand why, note that due to the randomness in the aDDM algorithm, two trials with identical stimuli and fixations might lead to different choices and RTs. As a result, two runs of the same trial can result in different outcomes even if they initially exhibit identical fixations. In addition, if the aDDM is an approximately accurate description of the underlying processes, the pattern of fixations can vary widely over repeated decisions with an identical pair of stimuli, a fact that is observed in the data. For these reasons, our out-of-sample predictions condition on the relative orientation of the stimuli, including the effect that this has on the fixation process, as described below, but not on the actual realized fixations. This allows us to test the ability of the aDDM to account out-of-sample for key patterns in the data conditional only on independent variables like the relative orientation of the stimuli.

For each of the 42 trial conditions we simulated 400 trials of the model, while sampling fixations, latencies and inter-fixation transitions from the empirical distributions, pooling the even-numbered trials from all subjects. Initial latency (i.e., the delay between stimulus appearance and the first item fixation) and subsequent inter-fixation transitions were sampled, each from its own distribution, without any further conditioning. To maximize the extent to which the simulated fixations matched the observed fixations, item fixations were sampled as follows. First, they were partitioned into 3 groups, corresponding to first, second and other middle fixations. Additionally, item fixations were conditioned on the relative proximity difference between the fixated and the unfixated items, *r*_*fixated*_ − *r*_*unfixated*_, since this matched the observed fixation patterns well. Note that the pool of fixations used to simulate the model excluded final fixations. According to the aDDM, a maximal fixation duration is drawn at the fixation outset and runs its course unless a barrier is cross beforehand. As a result, observed final fixations are truncated, and using them would bias the simulations (under the maintained hypothesis that the aDDM is correct).

Each trial was simulated by sampling latencies, fixations, and inter-fixation transitions as needed to carry out the simulation to its completion. In particular, each simulation began with a sampled latency, during which only white Gaussian noise was added to the RDV. Following this, fixations alternated between the left and right items such that, if the first fixated item was left, the second one would be right, and so on. The first fixation was chosen to be left with probability 0.65, which equals its empirical frequency. The maximum first fixation duration was sampled from the pool of first fixations, conditioned on *r*_*fixated*_ − *r*_*unfixated*_.

The simulation for a trial was terminated if the aDDM crossed a decision barrier during the course of a fixation. After each item fixation, an inter-fixation transition duration was sampled, and if a simulation happened to terminate on a transition, it was discarded, since this was not commonly observed in the data (mean percentage of trials across all subjects: 14.4%, min = 8.2%, max = 25.9%). We also simulated the model without discarding simulations that ended on transitions, but did not find any significant differences from the results presented here.

### Model comparisons

In order to explore the role of attention in explaining the data, we carried out an additional set of analyses designed to test the best fitting aDDM with the best fitting standard DDM, which equals the special case of θ = 1 where attention does not matter. To do this, we first re-estimated the model in the odd-number trials under the restriction that θ = 1 to find the best fitting standard DDM. We then carried out three different out-of-sample prediction exercises.

First, we predicted choices and RTs in the even trials using the best fitting DDM. This was done by simulating the model 100,000 times for each potential combination of *r*_*left*_ and *r*_*right*_, and then making predictions by sampling choices and RTs from the resulting simulations conditional on the stimulus orientations on each trial.

Second, we predicted choices and RTs in the even trials using the best fitting aDDM, conditional on net fixation time (i.e., total fixation time on left minus total fixation time on right). This was also done by simulating the model 100,000 times for each potential combination of *r*_*left*_ and *r*_*right*_, and then sampling choices and RTs from the resulting simulations, but this time conditional on both the stimulus orientations and the overall net fixation time observed in the even trial.

Third, we predicted choices and RTs in the even trials using the best fitting aDDM, and conditional on the observed fixations. To do this, we simulated the aDDM for each even trial assuming the same values of *r*_*left*_ and *r*_*right*_, and that the fixation process was identical to the one seen in the trial up to its RT. If the simulation did not lead to a choice by the observed RT, additional fixations were sampled using the fixation process described above. The outcomes of the simulation were used as the choice and RT predicted for each even trial.

### Goodness-of-fit measures

For binary variables, we report Efron's pseudo R-squared as a measure of goodness-of-fit, which corresponds to the squared correlation between the predicted values and the actual values. For non-binary variables, we report a number of goodness-of-fit measures, which are designed to test the similarity between the predicted and the observed data patterns. Each pattern involves a relationship between an independent (e.g., differences in relative proximity) and a dependent variable (e.g., RTs). Similar to previous work (Krajbich et al., 2010), these measures were computed as the *p*-values on the coefficients of a weighted least squares regression, in which the dependent variables were given by the difference between each subject's mean and the average value predicted by the model, and the weights were given by the inverse of the variance.

### Data and code

The data and code used in the analyses are available at the Rangel Neuroeconomics Lab website (www.rnl.caltech.edu).

## Results

In order to investigate the role of visual attention in perceptual decision-making, we carried out two different experiments. Experiment 1 was designed to test the extent to which the aDDM provides a reasonable quantitative description of the relationship between visual attention (as measured by fixations), choices and reaction times (RTs) in simple perceptual decisions. Experiment 2 was designed to test a key prediction of the aDDM; namely, that exogenous shifts in attention can bias perceptual decisions in favor of the attended item.

The first experiment, depicted in Figure 1, required subjects to make simple perceptual decisions about line orientations (see Materials and Methods for details). At the beginning of each block of trials, subjects were shown an oriented bar for 5 s, which served as the target for the entire block (Figure 1A). The orientation of the target was chosen from the set {20°, 35°, 55°, 70°}. In each decision trial subjects were shown two oriented bars, one on the left and one on the right, and had to decide which of them had an orientation closest to the target orientation by pressing a button (Figure 1B). The angular distance between each of the lines and the target, denoted by Δ, was chosen randomly from the set {−15°, −10°, −5°, 0°, 5°, 10°, 15°} (Figure 1C), with the constraint that the two stimuli could not have an equal orientation. Note that the correct response depends only on the angular distance, which is a relative orientation measure. For example, if $\Delta_{left}=-10^{o}$ and $\Delta_{right}=15^{o}$, the correct response is left, and if the two stimuli are equidistant to the target (e.g., if Δ_*left*_ = −10° and Δ_*right*_ = 10°, then either choice is considered correct. In order to motivate subjects to perform the task, a subset of the trials was selected at random at the end of the experiment and subjects earned $1 for each correct choice.

In order to familiarize the subjects with the stimuli, they also participated in a training task at the beginning of each block in which they were shown one oriented bar at a time and had to judge if it had the same orientation as the target (Figure 1D). Training was administered until a pre-specified performance criterion was reached on each block. See Materials and Methods for details.

### Perceptual aDDM

**Figure 3:**
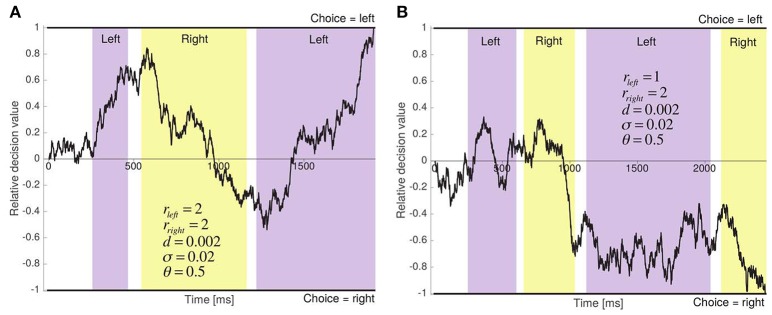
Two sample runs of the aDDM. **(A)** The two items have the same relative proximity, and the left item is chosen after 1,909 ms. **(B)** Right item has greater relative proximity, and is chosen after 2,444 ms.

The aDDM provides an algorithmic description of how information is integrated over time in order to make a binary perceptual choice, and of the role that fixations play in this process. As illustrated in Figure 3, the model assumes that choices are made by dynamically computing a relative decision value (RDV) signal, which at any instant provides an estimate of the relative attractiveness of the two options. The RDV begins at zero and a choice is made the first time it crosses one of two pre-established decision barriers: one at +1, indicating a choice for left, and one at −1, indicating a choice for right.

The predictions of the model depend heavily on the dynamics of the RDV. Let *RDV*_*t*_ denote its value at time *t* within the course of a single decision. At every time step Δ_*t*_, its change is given by μΔ_*t*_ + ε_*t*_, where ε_*t*_ is i.i.d. zero mean white Gaussian noise with standard deviation σ, and μ is the deterministic change in the RDV over the time step, often called the slope of the process. A critical assumption of the aDDM is that the slope of the RDV signal depends on the location of the fixations at each time step. In particular, μ = 0 until the first fixation to one of the two stimuli occurs, as well as during non-stimuli fixations and inter-fixation transitions, while μ = *d*(*r*_*left*_ − θ*r*_*right*_) during fixations to the left option, and μ = *d*(θ*r*_*left*_ − *r*_*right*_) during fixations to the right option. Here, *d* is a positive constant that controls the speed of integration, θ is a parameter between 0 and 1 that measures the size of the attentional bias, and *r*_*left*_ and *r*_*right*_ are the relative proximities of the left and right items shown in the trial.

The relative proximity of an option is a measure of its attractiveness, which in this task is given by the negative of the absolute value of Δ, and can only take four values: {−15°, −10°, −5°, 0°}. For ease of interpretation, and ease of comparison with related studies (Krajbich et al., 2010, 2012; Krajbich and Rangel, 2011), we normalized the relative proximities to the scale {0, 1, 2, 3}, with 3 denoting the best possible proximity (i.e., an orientation equal to the target), and 0 denoting the worst possible proximity (i.e., an angular distance of either −15° or +15°). We chose the range to be from 0 to 3 because there were 4 possible values for the angular distance between an item and the target. Figure S3 illustrates the transformation from the angular distance scale to the relative proximity scale.

The aDDM also makes a critical assumption about the fixation process. It allows fixations to depend on properties of the stimuli (as described below), but it assumes that the fixation process is otherwise independent of the state of the RDV signal. In other words, it assumes that there is no feedback from the path of the decision process to the propensity to fixate on stimuli. We return to this important assumption in the Discussion section.

No other major restrictions are placed on the fixation process, except for those that are reflected in the empirically observed properties of the fixations. First, the model assumes that the location of the first fixation, and its latency, are independent of the relative proximity of the two stimuli. To be precise, it assumes that the first fixation is to the left item with a constant probability *p*, and that the latency of this first fixation is drawn from a fixed distribution. Second, subsequent fixations alternate between the left and right items. Third, a maximal fixation duration is drawn from a distribution at the beginning of each fixation, and the fixations run their course unless a choice is made by crossing a barrier before the end of the fixation. In this case, the process terminates and the duration of the last fixation is truncated. The distribution of maximal fixation durations is allowed to depend on the fixation number, and on the difference in relative orientation between the fixated and the unfixated stimuli (see Materials and Methods for details). Importantly, we only sample from non-last fixations, i.e., fixations that were not terminated when the subject makes a choice. Fourth, fixations are separated by inter-fixation transitions that are drawn from another fixed distribution. As with the fixations, a maximal inter-fixation transition duration is drawn from this distribution at the beginning of each transition, and runs its course unless a barrier is crossed before it terminates.

Several aspects of the model are worth highlighting. First, the model has three free parameters: *d*, θ, and σ (the time step used for binning the data was fixed at 10 ms). This follows from the fact that, in this class of models, multiplying the size of the barriers, *d* and σ by a common positive constant does not change the predictions of the model. As a result, we can fix the size of the barriers to +1 and −1 without any loss of generality. Second, if θ = 1, the model reduces to the standard drift-diffusion model (DDM) (Ratcliff, 1978; Gold and Shadlen, 2002, 2007; Ratcliff et al., 2003; Ratcliff and Smith, 2004; Smith and Ratcliff, 2004; Bogacz, 2007; Ratcliff and Mckoon, 2008) and therefore item fixations become irrelevant. Thus, the model includes as a special case the possibility that attention plays no role in choices. Third, if θ < 1, the model predicts that changes in fixations can affect choices. The intuition for why this is the case is illustrated in Figure 3A, which depicts a sample run in which r_*left*_ = r_*right*_ = 2. In the absence of an attentional bias (i.e., when θ = 1), the mean slope of the RDV signal is zero and the choice and RT are determined solely by the noise in the process. In contrast, when θ = 0.5, as shown in the figure, the mean slope of the RDV signal is positive during left fixations and negative during right fixations (i.e., the integrator moves toward the fixated item on average). Fourth, when θ < 1, exogenous shifts (i.e., unrelated to the perceptual properties of the stimuli) in fixations toward an item can bias choices toward that item, and the magnitude of the bias increases as θ decreases. For instance, as shown in Figure 3B, when r_*left*_ < r_*right*_ and θ = 0.5, the attentional bias strengthens the negative slope toward the right item barrier during right fixations. Fifth, the assumption that the fixation process does not depend on the state of the RDV signal implies that one can think of the aDDM as a model of the decision process that takes as given the empirical relationship between fixations and various non-aDDM variables (e.g., fixation number or relative proximity), presumably because the choices and fixations are controlled by distinct systems.

### Basic psychometrics

**Figure 4:**
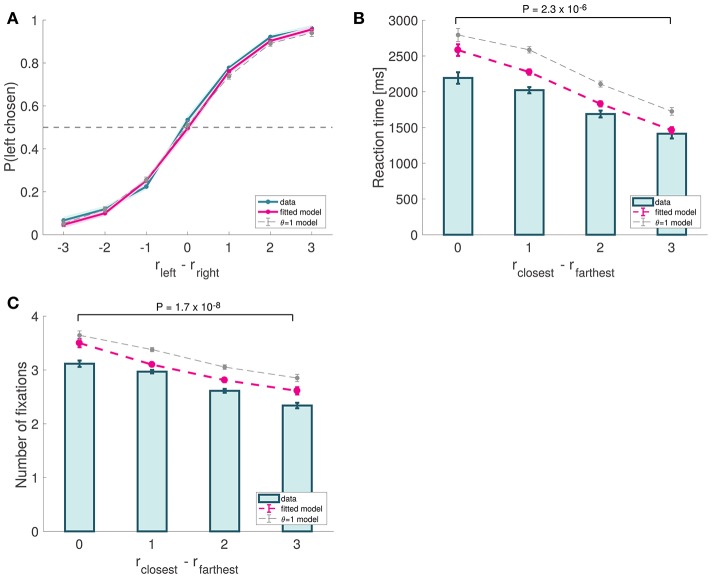
Basic psychometrics for Experiment 1. **(A)** Psychometric choice curve. **(B)** RT curve depicting mean response times vs. trial ease, as measured by the difference in absolute proximity between the correct and incorrect options. **(C)** Mean number of fixations vs. trial ease. Subject data includes only even-numbered trials. Fitted model is the best fitting aDDM with free θ, and θ = 1 corresponds to the DDM. Error bars show 95% confidence intervals for the data pooled across all subjects, and across all simulated trials in the case of the data predicted by the models. Tests are based on a paired two-sided *t*-test.

We began the analysis by characterizing the basic psychometrics of the task, which resembled the patterns commonly found in previous perceptual (Ratcliff et al., 2003, 2009; Churchland et al., 2008; Deco et al., 2010; Bode et al., 2012; Bowman et al., 2012; Van Vugt et al., 2012; White et al., 2012; Brunton et al., 2013; Ossmy et al., 2013) and value-based decision-making studies (Gold and Shadlen, 2007; Krajbich et al., 2010, 2012; Mormann et al., 2010; Krajbich and Rangel, 2011; Hunt et al., 2012; Tsetsos et al., 2012a; Philiastides and Ratcliff, 2013). Choices were well described by a logistic function of the relative attractiveness of the two items with a significant but negligible bias (mixed effects logistic regression: constant = 0.08246, *p* = 0.0115, slope = 1.15047, *P* < 10^−16^; Figure 4A). The mean frequency of correct trials across subjects was 86.3% (*SD* = 5.1%). Reaction times decreased as choice ease increased (mixed effects linear regression: slope = −277.77 ms, *p* = 10^−11^; Figure 4B). We measured choice ease using the relative proximity difference between the items with the closest and farthest orientations to the orientation of the target. The mean reaction time was 1,849 ms (*SD* = 613 ms). Also consistent with previous studies (Krajbich et al., 2010; Krajbich and Rangel, 2011), we found that the number of fixations per trial decreased as choice ease increased (mixed effects linear regression: slope = −0.28 fixations, *p* = 10^−20^; Figure 4C). The mean number of fixations was 2.83 (*SD* = 0.39). Together, these analyses showed that our perceptual task exhibits psychometric properties common in 2-alternative forced choice tasks, which are predicted by a wide class of sequential integrator models, including the aDDM.

### Properties of fixations

We recorded fixations using an eye-tracker, which allowed us to characterize their properties during the choice process. For this purpose, we classified each item fixation as “first,” “middle” or “last,” according to when it occurred within the trial. “Middle” fixations are those that are neither the first nor the last ones.

**Figure 5:**
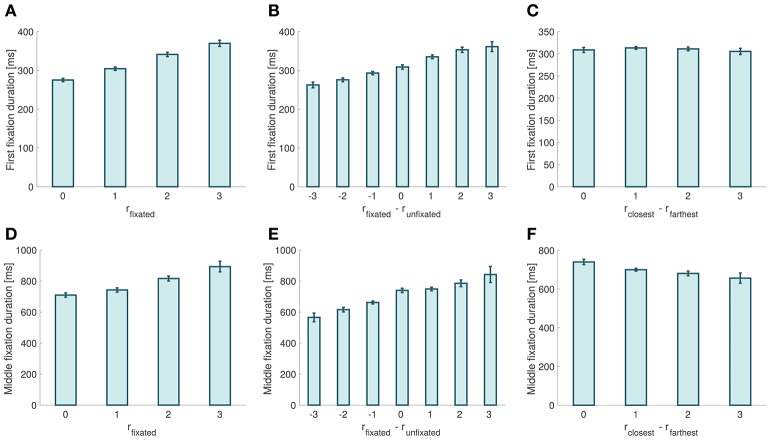
Fixation properties. **(A)** First fixation duration as a function of the relative proximity of the fixated item. **(B)** First fixation duration as a function of the relative proximity difference between the fixated and the unfixated items. **(C)** First fixation duration as a function of choice ease. **(D)** Middle fixation duration as a function of the relative proximity of the fixated item. **(E)** Middle fixation duration as a function of the relative proximity difference between the fixated and the unfixated items. **(F)** Middle fixation duration as a function of choice ease. Error bars show 95% confidence intervals for the data pooled across all subjects.

We found that the probability that the first fixation is to the item with closest orientation to the target was not significantly different from chance, and was independent of the relative proximity difference between the two stimuli (mixed effects linear regression: slope = −0.009, *p* = 0.095). Moreover, we found that the duration of first fixations increased with the relative proximity of the fixated item (mixed effects linear regression: slope = 30.55 ms, *p* = 10^−12^; Figure 5A), decreased with the relative proximity of the unfixated item (mixed effects liner regression: slope = −11.36 ms, *p* = 10^−7^), and increased with the relative proximity difference between fixated and unfixated items (mixed effects linear regression: slope = 17.67 ms, *p* = 10^−14^; Figure 5B). We did not find a significant correlation between first fixation durations and choice ease (mixed effects linear regression: slope = −1.36 ms, *p* = 0.55; Figure 5C).

When looking at middle fixations, we found that their duration increased with the relative proximity of the fixated item (mixed effects linear regression: slope = 59.9 ms, *p* = 10^−17^; Figure 5D), and decreased with the relative proximity of the unfixated item (mixed effects linear regression: slope = −40.187 ms, *p* = 10^−5^). Middle fixation durations also increased with the relative proximity difference between the fixated and the unfixated items (mixed effects linear regression: slope = 40.77 ms, *p* = 10^−21^; Figure 5E). Finally, we found that middle fixation durations decreased as choices became easier (mixed effects linear regression: slope = −24.4 ms, *p* = 0.00075; Figure 5F).

These findings show that the observed fixations patterns are consistent with the assumptions of the aDDM described above. Importantly, note that these analyses did not include last fixations because their duration is endogenous in the aDDM, even under the maintained hypothesis that fixation durations and locations are not affected by the state of the choice process. The endogeneity of the last fixations follows from the simple fact that they are terminated whenever the choice is made upon crossing a barrier.

### Model fitting

We divided the data into even- and odd-numbered trials, used the odd trials to fit the free parameters of the aDDM using maximum likelihood estimation (see Materials and Methods for details), and then tested the predictions of the model out-of-sample using the even trials. The best fitting parameters resulting from the group-level MLE were *d* = 0.0041, σ = 0.063 and θ = 0.36 (using a time step of size 10 ms). Since θ is much smaller than 1, this suggests a sizable attentional bias.

We then used the best fitting parameters to simulate behavior in the even-trials, and compared it to the actual observed data (see Materials and Methods for details). The simulated data provided a reasonably good qualitative and quantitative match to the observed out-of-sample behavior. The psychometric choice curve (Efron's pseudo *R*^2^ = 0.086; Figure 4A) predicted that choices are a logistic function of relative orientation differences, and that reaction times (goodness-of-fit: *p* = 0.10, Figure 4B) and number of fixations (goodness-of-fit: *p* = 0.22, Figure 4C) decrease as choice ease increases.

In the Supplementary Materials we present three additional sets of results that might be of interest to the reader. First, we provide individual subject fits. Second, we estimate a non-linear version of the aDDM and find that the best fitting model is approximately linear (as in the basic aDDM). Third, we fit the aDDM separately for trials in the first half of each block, where the memory of the target orientation is fresh, and trials in the second half of the block, where the memory of the target orientation might have dissipated. The best fitting parameters in both cases are very similar, which suggests that this was not an issue affecting performance in the task.

### Model predictions

We next tested for several basic predictions of the aDDM.

**Figure 6:**
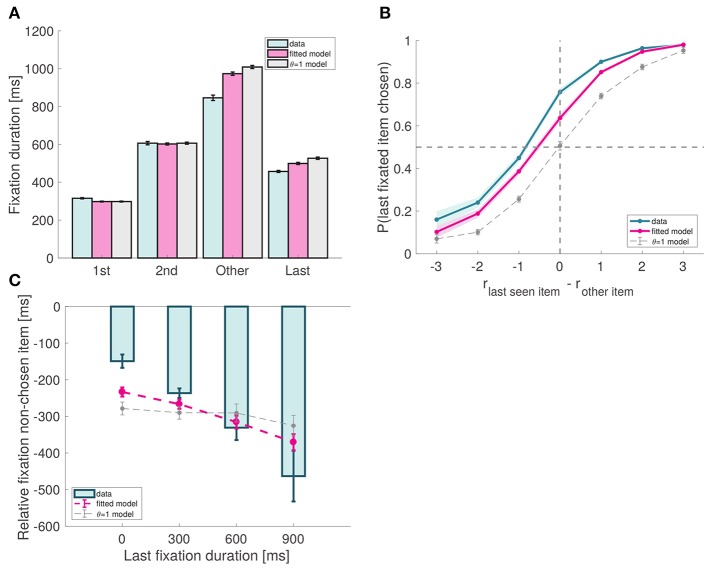
Model predictions. **(A)** Fixation duration by type. As predicted by the model, last fixations are shorter than middle fixations. Note that, except for last fixations, the match between the data and the model is a direct consequence of our fixation sampling process. **(B)** Probability that the last fixation is to the chosen item as a function of the relative proximity difference between the last fixated item and the other item. In the absence of a bias effect, the probability at 0 should be around 0.5; due to the bias, the observed probability is significantly larger than 0.5. **(C)** Amount of time spent looking more at item A before the last fixation (to item B), as a function of the duration of that last fixation. Subject data includes only even-numbered trials. Fitted model is the best fitting aDDM with free θ, and θ = 1 corresponds to the DDM. Error bars show 95% confidence intervals for the data pooled across all subjects, and across all simulated trials in the case of the models.

First, the model predicts that final fixations should be shorter than middle fixations. This prediction follows from the fact that, according to the model, last fixations are interrupted when the RDV reaches one of the barriers, cutting the last fixation short. We found this to be the case in our data, as both second fixations as well as other middle fixations (middle fixations excluding second fixations) are significantly longer than last fixations (*p* = 10^−9^ and *p* = 10^−15^, respectively; Figure 6A).

Second, the model predicts that subjects should exhibit a bias toward choosing the last fixated item, even in trials where they have fixated on both of them. This prediction follows from the fact that when θ < 1, the RDV moves toward the decision barrier of the fixated item unless it is significantly less desirable than the other item. For example, when θ = 0.5, the RDV moves toward the left barrier when fixating left as long as r_*left*_ > 0.5 r_*right*_. This pattern was observed both in the data and the simulations (Efron's pseudo *R*^2^ = 0.11; Figure 6B).

Third, the model predicts a very specific relationship between the duration of the last fixation and the pattern of previous fixations. At any point in time within the trial, we can compute the relative fixation time of the fixated item, given by the total fixation time on that item thus far minus the total fixation time on the other item thus far. The model predicts that in trials where the last fixated item is chosen, the duration of the last fixation decreases with its relative fixation time computed at the beginning of the last fixation. This effect is due to the nature of the RDV signal: when θ < 1, the longer an item is fixated in the trial, the more the signal will move toward its barrier, so the last fixation to the other item (which will eventually be chosen) will have to be longer so that the signal can move back toward its barrier. As shown in Figure 6C, this effect was present in the data (mixed effects linear regression: slope = −0.28, *p* = 10^−11^) and the simulations (goodness-of-fit: *p* = 0.48).

### Choice biases

The aDDM also predicts several choice biases when θ < 1, which we tested next.

**Figure 7:**
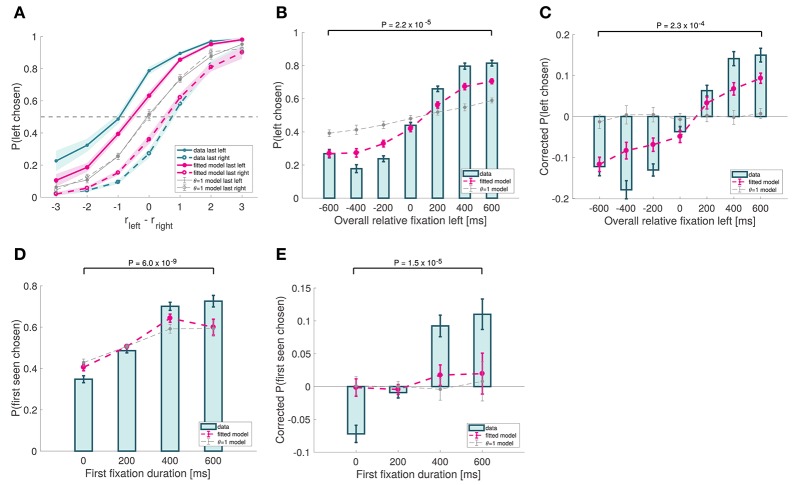
Choice biases. **(A)** Psychometric choice curves conditioned on the location of the last fixation. **(B)** Probability that the left item is chosen as a function of the excess amount of time for which the left item was fixated during the trial. **(C)** Analogous to **(B)**, except subtracting the average probability of choosing left for each relative proximity difference. **(D)** Probability that the first-seen item is chosen as a function of the duration of that first fixation. **(E)** Analogous to **(D)**, except subtracting the average probability of choosing the first-seen item for each relative proximity difference. Subject data includes only even-numbered trials. Fitted model is the best fitting aDDM with free θ, and θ = 1 corresponds to the DDM. Error bars show 95% confidence intervals for the data pooled across all subjects, and across all simulated trials in the case of the models. Tests are based on a paired two-sided *t*-test.

First, the model correctly predicts a last-fixation bias: subjects are more likely to choose the last item fixated in the trial (Efron's pseudo *R*^2^ = 0.11 for left last fixated, and 0.084 for right last fixated; Figure 7A), due to the fact that the relative proximity of the unfixated item is underweighted in the RDV integration process. Note that a sizeable bias effect can be seen both in our data as well as in the simulations (for instance, when *r*_*left*_ − *r*_*right*_ = 0, the difference in the probability of choosing left when left was last fixated vs. when right was last fixated is 0.51 for the data, and 0.26 for the simulations).

Second, the model predicts that the probability of choosing an item increases with its overall relative fixation time. This follows from the fact that, because the RDV moves toward the barrier of the fixated item (unless it is significantly worse than the other one), the RDV is more likely to move in the direction of an item's barrier when that item is being fixated than when it is not. Consistent with this, we found a strong association between overall relative fixation times and choices (Efron's pseudo *R*^2^ = 0.14; Figure 7B). However, a concern with this test is that overall relative fixation times and relative proximity are correlated. To correct for this, we computed a corrected choice probability curve by subtracting from each trial's choice (1 for left and 0 for right) the average probability of choosing left for that particular relative proximity difference. This curve provides an uncontaminated measure of the effect of relative fixation at the time of choice, under the assumptions of the aDDM. As shown in Figure 7C, the observed choice bias was sizable, and matched well the simulated data (goodness-of-fit: *p* = 0.038).

Finally, the model also predicts that the likelihood of choosing the first seen item increases with the duration of the first fixation. This was observed in the data (mixed effects linear regression: slope = 0.00075, *p* = 10^−35^; Figure 7D) and in the simulations (Efron's pseudo *R*^2^ = 0.088). The effect was still present after correcting for relative proximity differences, by subtracting from each trial's choice the average probability of choosing the first-seen item for that particular relative proximity difference (goodness-of-fit: *p* = 0.021; Figure 7E).

### Model comparison

As discussed above, the aDDM reduces to the standard DDM, in which fixations do not affect choices or RTs, when θ = 1. This provides an additional way of exploring the role of visual attention, by comparing the ability of the best fitting aDDM and the best fitting DDM to explain the data out-of-sample.

This was done in several steps. First, we re-estimated the model in the odd-numbered trials under the restriction that θ = 1, which amounts to finding the best fitting DDM. We found that the best fitting parameters in this case were *d* = 0.0024 and σ = 0.062. In contrast, the best fitting parameters for the aDDM were *d* = 0.0041, σ = 0.063, and θ = 0.36.

Second, we used these best fitting parameters to carry out the same out-of-sample predictions described above, but for the best fitting DDM. As shown throughout the figures, the results show that when θ = 1 the model cannot account for key aspects of the data patterns and choice biases. For example, consider Figure 6B, which shows that there is a sizable choice bias in favor of the last fixation. As the figure shows, the best fitting aDDM can account for this pattern, which follows from the overweighting of *r*_*fixated*_ relative to *r*_*unfixated*_. In contrast, the best fitting standard DDM cannot explain this pattern since attention does not matter in that model.

Third, we compared the ability of the two models to predict choices and RTs out-of-sample, which provides a test of the value of fixation data in predicting choices and RTs (see Materials and Methods for details). We compared the accuracy of three types of predictions. For the standard DDM we predicted choices and RTs in the even-numbered trials, conditional only on *r*_*left*_ and *r*_*right*_, since fixations do not matter in this case. For the aDDM we carried out two different sets of predictions. In one of them we predicted choices and RTs, conditional on *r*_*left*_, *r*_*right*_ and on the net fixation time on the left item (i.e., total fixation time on left minus total fixation time on right) in each even trial. In the other, we made predictions based on *r*_*left*_, *r*_*right*_ and, as much as possible, on the actual path of fixations observed on each trial. The most accurate predictions were made by the best fitting aDDM conditional on net fixation time (choice prediction accuracy = 72.3%, average RT absolute error = 1.92 s). The second most accurate predictions were made by the other aDDM exercise (choice prediction accuracy = 70.1%, average RT absolute error = 2.49 s). The least accurate predictions were made by the best fitting DDM (choice prediction accuracy = 68.4%, average RT absolute error = 2.69 s). To put these numbers in perspective, note that the standard deviation of RTs is 1.87s. Together, these results suggest that incorporating fixation information in the way specified by the aDDM improves the out-of-sample predictions made by this class of models.

It might seem counter intuitive that predictions that condition on the observed fixations as much as possible perform worse than those that condition only on observed net fixation time. However, this makes sense once the randomness in the aDDM model is taken into account. Since the model entails significant randomness, repeated runs of the model with the same stimuli will result on different choices, RTs, and net fixation times, even if they follow the same fixation pattern as much as possible. As a result, repeated runs of the exact same trials can result in net fixations times that are significantly different from those observed in the trial that we are trying to predict, even if the repeated runs require that fixations follow the same process as long as possible (see Section Materials and Methods for details). In contrast, the other set of aDDM predictions are made using runs of the aDDM that result in nearly identical net fixation times. This leads to better predictions because net fixation time on the two items affects the average slope of integration in the diffusion model, and thus choices and RTs. Consistent with this, the standard deviation in the simulated net fixation times was 276 ms in the first aDDM prediction model, 886 ms in the second aDDM prediction model, and 2,603 ms in the DDM prediction model.

### Experiment 2: causal test of the attentional effect

So far we have found that the aDDM provides a reasonable quantitative and qualitative account of the relationship between fixations, choices and RTs in our perceptual task. Importantly, while the aDDM predicts a causal impact of attention on perceptual choice, the evidence presented so far is only correlational. We addressed this issue using an experimental paradigm that manipulates item fixation times with the aid of an eye-tracker, and which has been previously shown to causally affect subjects' choices on a moral decision task (Pärnamets et al., 2015).

The task, depicted in Figure 2, consisted of a modification of the previously described perceptual choice task. The key difference is that in each trial we randomly selected one of the two items on the screen to be the bias-target for that trial, and implemented the following procedure to bias fixation toward that item (see Materials and Methods for details). Unbeknownst to the subjects, we defined a minimum period of time required for them to fixate on each item before a decision could be made: 800 ms for the bias-target and 200 ms for the non-bias-target. We then used the eye-tracker to record the duration of each fixation and, as soon as the minimum requirement for both items was met, the items disappeared from the screen, and the subject was asked to make a choice. Note that this requirement does not guarantee that the bias-target will be fixated longer, since it only establishes a minimum amount of time for each item to be fixated, but not a maximum amount. To ensure that subjects would not become aware of our manipulation, we set the maximum duration for each decision trial at 3 s. If the minimum fixation requirements for both items were not met within that period, the subject was prompted to make a decision, and the trial would be discarded from all future analyses. Overall, 24.3% of the trials (1,633 trials) were discarded in this manner. We refer to trials in which the manipulation was effective (i.e., in which the bias-target was fixated longer) as *effective trials*. In order to increase the number of effective trials, we also attempted to guide subjects' first item fixations toward the bias-target (Hikosaka et al., 1993), so that it would have a better chance of being fixated longer. In particular, the bias-target stimulus was always displayed first, and the other stimulus appeared on the screen after a short delay (duration: mean = 450 ms, *SD* = 35 ms). Using this manipulation, 74.3% of the non-discarded trials were effective (i.e., a total of 3780 trials were effective).

**Figure 8:**
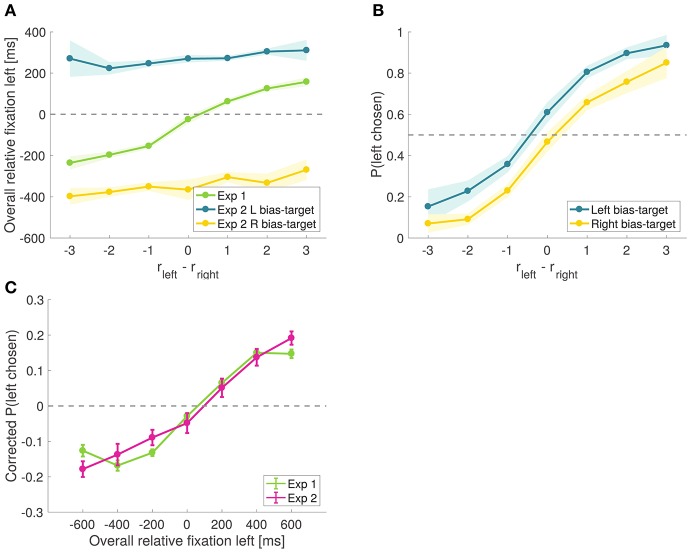
Causal test of the attentional effect. **(A)** Time advantage of the left item over the right item comparing effective trials from Experiment 2 to all trials from Experiment 1. **(B)** Psychometric choice curves conditioned on the bias-target item. Subjects choose the bias-target with higher probability. Shaded error bars show 95% confidence intervals for the data pooled across all subjects. **(C)** Corrected probability that the left item is chosen as a function of the excess amount of time for which the left item was fixated during the trial, comparing all trials from Experiment 1 to all trials from Experiment 2. Corrected probabilities are obtained by subtracting from each trial's choice (1 for left and 0 for right) the average probability of choosing left for the relative proximity difference from that trial.

To check the success of the experimental manipulation, we compared the overall relative fixation time advantage of the left item in all trials from Experiment 1 vs. all effective trials in Experiment 2. As shown in Figure 8A, in Experiment 1 the left time advantage increased with the difference in relative proximity, while in Experiment 2 the bias-target was fixated longer regardless of the items' relative proximity. In effective trials, the mean total fixation time on the bias-target was 814.2 ± 85.8 ms, while for the other item it was 509.2 ± 46.1 ms.

As predicted by the aDDM, we found that the probability of choosing the left item was higher on trials where the left item was the bias-target than on those where the right item was the bias-target (Figure 8B). To appreciate the magnitude of the bias, note that when *r*_*left*_ − *r*_*right*_ = 0, the probability of choosing left increases by 14% across the two conditions (χ^2^ statistic = 16.51, *p* = 10^−5^).

Importantly, the magnitude of the bias is comparable in magnitude to what we found in Experiment 1. In particular, the slope of the corrected choice curve in Figure 7C is ~0.02, which implies that a shift in relative fixation of 300 ms (which is similar in size to the one induced by the experimental manipulation in Experiment 2) should induce about a 6% increase in the probability of choosing the item. Figure 8C shows a comparison of this effect between Experiments 1 and 2, illustrating that the quantitative effects of the causal manipulation in Experiment 2 are consistent with the measurements from Experiment 1, providing additional support for the validity of the causal manipulation.

**Figure 9:**
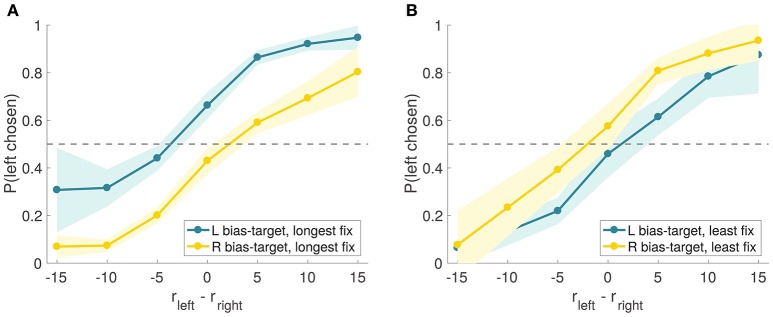
Experiment 2 choice curves. Experiment 2 psychometric choice curves conditioned on the bias-target item and on whether the bias-target was the longest **(A)** or least **(B)** fixated item in the trial (as measured by the total fixation time on each item).

A natural concern with these results is that the observed effect might have been due, in part, to priming: since the first fixation was manipulated to be to the bias-target item, this could have primed the subjects to bias their choices in this direction (Meyer and Schvaneveldt, 1971; Nedungadi, 1990). Another concern is that, by discarding trials in which the manipulation was not effective, we are biasing the results toward the hypothesis that longer fixations increase the probability of choosing the fixated option. To address these issues, we split the trials into two sets based on whether or not the bias-target was the longest fixated item in the trial (i.e., effective vs. ineffective trials), regardless of which item was fixated first, and compared the size of the bias in these two types of trials (Figure 9). If the observed effect were exclusively due to priming, one would expect a similar choice bias in both groups of trials. In contrast, the aDDM predicts a stronger choice bias in the trials in which the bias-target was fixated longer. Consistent with this, we found that the choice-bias was larger in trials where the bias-target was fixated longer than in those when it was fixated less (comparison of the individual biases in logit regressions: mean constant difference 1.45 vs. 0.57, paired *t*-test *t* = 7.13 and *p* = 10^−8^ vs. *t* = 0.27 and *p* = 0.79; when the bias-target was longest fixated, Figure 9A, mean total fixation time was 814 ms for the bias-target and 509 ms for the non-bias-target, and when the bias-target was least fixated, Figure 9B, mean total fixation time was 801 ms for the bias-target and 1,132 ms for the non-bias-target).

## Discussion

The results described above provide evidence consistent with the hypothesis that the aDDM gives a plausible algorithmic description of the impact of attention in simple perceptual decision-making. Experiment 1 shows that the model is able to provide a reasonably good (although not perfect) quantitative description of the relationship between fluctuations in visual attention, choices and reaction times. Experiment 2 shows that the impact of attention in choice predicted by the aDDM is causal, and of a qualitatively similar size as that predicted by the best fitting model in Experiment 1.

The imperfect match between our data and the model simulations could be due to at least two important factors. First, the model we used to simulate the fixation process is a simplistic approximation, which, under the assumptions of the aDDM, adds noise to the simulations. Second, the results presented for the model simulations are averaged across trials, so the fact that they are not conditioned on the same fixations present in the data also adds noise.

The version of the aDDM tested here is virtually identical to the one used in previous value-based choice studies (Krajbich et al., 2010, 2012; Krajbich and Rangel, 2011; Towal et al., 2013). The only difference is that in value-based choice the evidence that is integrated is composed of noisy measurements of preference for the stimuli, whereas here it is noisy perceptual signals about line orientation. This suggests that a similar simple class of algorithms with only three free parameters is able to provide a quantitative characterization of several complex behavioral patterns in the data, such as the impact of relative fixation durations, or the impact of first fixations. This provides further support for the view that the brain utilizes similar algorithms, and perhaps similar neural architectures, for sufficiently similar classes of problems, even if they operate in domains as different as perception and value-based choice.

Suggestively, the attentional bias we found in this study (θ = 0.36) is substantial and of similar size to the bias found in previous value-based studies (θ = 0.3) (Krajbich et al., 2010). This result leads us to speculate that attentional biases might be sizable in any simple decision task (perceptual or value-based) in which fixations facilitate the evidence gathering process.

An important feature of the aDDM is that it posits a causal impact of attention on choice. In particular, it assumes that the evidence related to fixated items is weighted more heavily during the decision process, and as a result choices can be biased toward a stimulus by increasing its share of fixations. Furthermore, when the attentional bias parameter is much smaller than 1, the predicted biases can be sizable. Previous studies of value-based choice with exogenously manipulated fixations have found causal effects in the predicted direction, but of smaller magnitude than predicted by the model (Shimojo et al., 2003; Armel et al., 2008). One potential explanation for the small effect sizes is that the experimental manipulations had limited success in shifting attention. Experiment 2 provides evidence consistent with this interpretation. Here we utilized a different experimental manipulation of attention that was able to shift fixations and found a causal effect of a similar magnitude as the one predicted by the model. In fact, our attentional manipulation was inspired by a recent study of attention and moral decisions, which also found sizable effects (Pärnamets et al., 2015).

A critical assumption of the aDDM is that the fixation process is independent from the sequential integration process. More precisely, random fluctuations in the RDV variable that guides the choices do not affect the fixation process. We emphasize that this does not rule out the possibility that stimulus properties orthogonal to the decision process might affect fixations. In fact, a recent study in the domain of value-based choice showed that the fixation process was affected by low level visual features (Towal et al., 2013), and several others have provided evidence that the value integration process is modulated by the saliency of the stimuli (Tsetsos et al., 2012a, 2016). Instead, the key assumption of the model is that the actual integration process does not affect the fixation process. Thus, the aDDM can be thought of as a model of the decision process, taking as given the exogenous and potentially stochastic fixation process. In this study, as well as previous ones (Krajbich et al., 2010; Krajbich and Rangel, 2011; Towal et al., 2013), this is implemented by taking the fixation process to be the one that best describes the one observed in the data.

We do not view our results as providing evidence for the hypothesis that the attentional process is not influenced by the state of the decision process variables. In fact, studies of value-based choice with large numbers of items have found that fixations are shifted toward the best items several seconds into the decision process (Reutskaja et al., 2011). Instead, our results suggest that these additional influences on attention have a limited impact on the choice process, since most of the effects are already accounted for by the simpler aDDM. However, we also conjecture that “top-down” modulations of attention are more likely to occur in more complex decisions with longer reaction times. A full characterization of how the decision process affects attention, and how this feeds back to the choices, is a critical open question for ongoing research. For example, a recent study has shown that some of the choice bias toward the last fixated item shown here can arise in multiple types of integrator models, even when attention is entirely random and independent of the choice process (Reutskaja et al., 2011; Mullett and Stewart, 2016).

The aDDM provides a simple way of introducing attention in sequential integrator models of choice, by adding an extra parameter to the most basic version of the Drift Diffusion Model. However, similar modifications could be introduced to a number of other sequential integrator models of choice, including leaky-accumulator models (Usher and Mcclelland, 2001), neural network models of the choice process (Wong and Wang, 2006; Hunt et al., 2012), or more complex versions of the Drift Diffusion Model (Churchland et al., 2008; Ratcliff and Mckoon, 2008; Mormann et al., 2011; Hawkins et al., 2015), among others. Such modifications would have qualitatively similar effects, provided that the assumption that fixations are orthogonal to the state of the decision process is maintained. Given the active debate in the literature about which sequential integrator model provides the best description of the underlying processes, an important direction for future research is to carry out a systematic comparison of the attentional versions of all of these models.

The aDDM models the effects of attention at a high level of abstraction. Another important question for future research is to characterize the neural mechanisms behind the attentional effects captured by the model. For example, does attention operate at the perceptual stage, prior to the integration of the information by the decision process, or does it operate in the decision process itself? Does attention operate through similar channels in perceptual and value-based choice, and if so, why does it have a similar effect on choice?

## Ethics statement

This study was carried out in accordance with the recommendations of the Caltech IRB with written informed consent from all subjects. All subjects gave written informed consent in accordance with the Declaration of Helsinki. The protocol was approved by the Caltech IRB.

## Author contributions

GT, PP, and AR designed the experiment, performed the data analysis and wrote the manuscript. GT collected the data.

### Conflict of interest statement

The authors declare that the research was conducted in the absence of any commercial or financial relationships that could be construed as a potential conflict of interest.
