# Supplementary material for: The Attentional Drift Diffusion Model of Simple Perceptual Decision-Making
Source: Front Neurosci. 2017 Aug 24;11:468. doi: 10.3389/fnins.2017.00468 (PMC5573732; doi:10.3389/fnins.2017.00468)

The attentional Drift Diffusion Model of simple perceptual decision-making

SUPPLEMENTARY MATERIALS

Gabriela Tavares[1,*], Pietro Perona[1] and Antonio Rangel[1,2]
[1]Computation and Neural Systems, California Institute of Technology, Pasadena, CA, USA. [2]Division of Humanities and Social Sciences, California Institute of Technology, Pasadena, CA, USA. [*]Correspondence: gtavares@caltech.edu

INDEX

A. Likelihood computation: Details.
B. Robustness: additional tests for Experiment 1.
C. Individual subject fits for Experiment 1.

A. Likelihood computation: details

We used the following simulation procedure to compute the likelihood of the experimental data under the aDDM. For each set of parameters, we calculated the likelihood of the observed choice and reaction time (RT), conditional on the fixations observed in the trial.

The simulations were carried out using a discretized state space: time evolved in steps of 10 ms, and the RDV was discretized into 21 bins of size 0.1. As illustrated in **Figure S1**, this transforms the state-space of the model into a two-dimensional table. Columns denote time, increasing from left to right. Rows denote the state of the RDV.

At any discretized time point, the simulation assigns a probability mass to each RDV bin, which measures the likelihood that the value of the RDV signal falls in that bin, at that time, conditional on a decision not having made yet (because a decision barrier has not been crossed). Importantly, note that the sum of these likelihoods in a particular RT column need not add to 1 if there is a positive probability that a decision can be made before that time.

The simulation starts at time zero (first column of the table) with the entire probability of the RDV signal placed in the zero-bin with probability 1. This reflects the assumption that in the aDDM the value of the RDV is zero at the beginning of every decision.

We then fill out the columns of the table from left to right, as illustrated in **Figure S2**. Let P_t^i denote the probability of bin i at time step t. For any RDV bin j between the barriers, the likelihood of the model being in RDV bin j at time $t+1$ is given by

$$P_{t+1}^{j} = \sum_{i} P_t^i \times P_t^{i\to j}$$

where $P_t^{i\to j}$ denotes the probability of transition from i to j. This transition probability is easily computed from the equations describing the aDDM. The change in the RDV in a single time step is given by $N(\mu,\sigma)$, with $\mu = 0$ before the first fixation to an item occurs, during non-item fixations and during inter-fixation transitions. During item fixations, $\mu = d(r_{left} - \theta r_{right})$ for fixations to the left option, and $\mu = d(\theta r_{left} - r_{right})$ for fixations to the right option. The probability of transition $P_t^{i\to j}$ is therefore given by the value of the probability density function $N(\mu,\sigma)$ for δ, where δ is the difference in mean RDV values between bins j and i.

In order to ensure that the fixation state is fixed throughout the duration of each time bin, we take the duration of each fixation in milliseconds and divide it by the size of the time bin (10 ms). We then discard the remainder, such that each corrected fixation duration is now an exact multiple of the time bin size. By doing this, we simplify the computation by having all fixations terminate at the end of a time bin.

At every time step, we also calculate the probability of the RDV reaching each of the two barriers. In particular, the likelihood of the model crossing the upper barrier at time step $t+1$ is given by

$$P_{t+1}^{UP} = \sum_{i} P_t^i \times P_t^{i \to UP}$$

where $P_t^{i \to UP}$ denotes the probability of going from a value of RDV in bin i to crossing the upper barrier at time step t, and is given by the probability of a draw from $N(\mu, \sigma)$ that exceeds $1 - i$. P_{t+1}^{DOWN} is defined analogously.

For every trial, the model is simulated by filling out the table until the observed RT is reached. The likelihood for that trial is then given by P_{RT}^{UP}, if the individual chose left, and by P_{RT}^{DOWN}, if the individual chose right.

The process is illustrated in **Figure S2**, in which the barrier crossing probabilities are depicted as additional top and bottom rows shaded in gray.

Figure S1. Discretization of the aDDM state space.

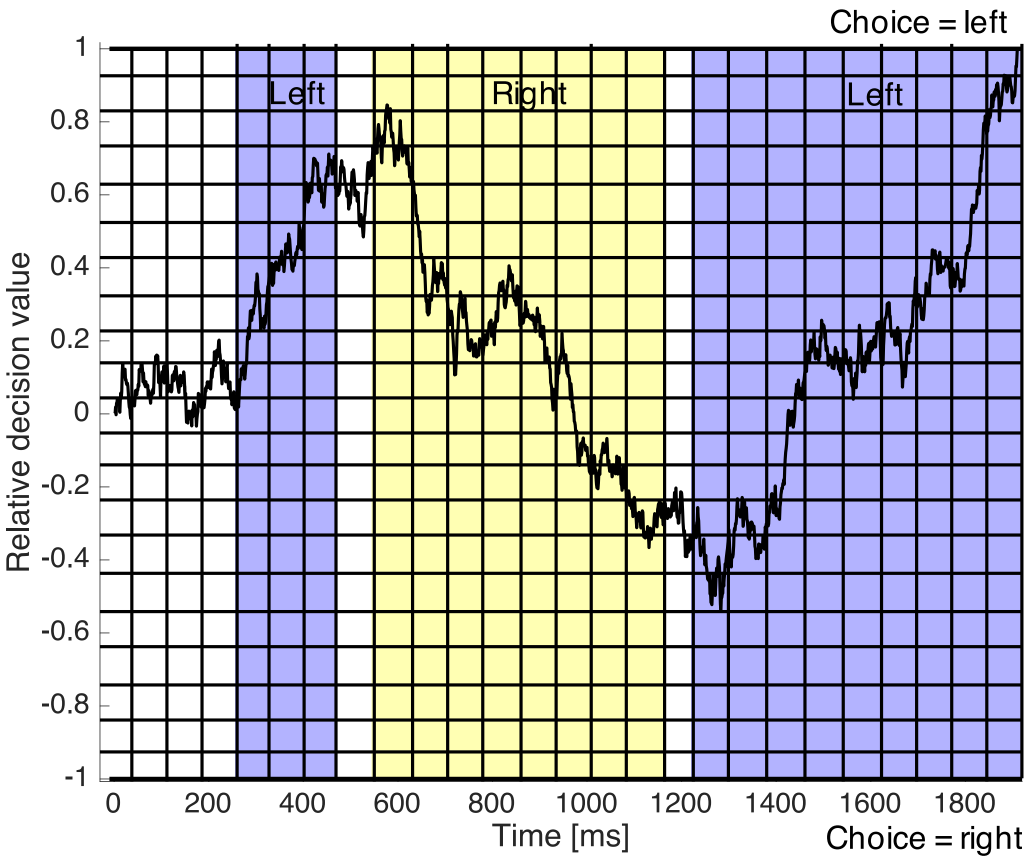

Figure S2. Iterative computation of the likelihood in the discretized state space.

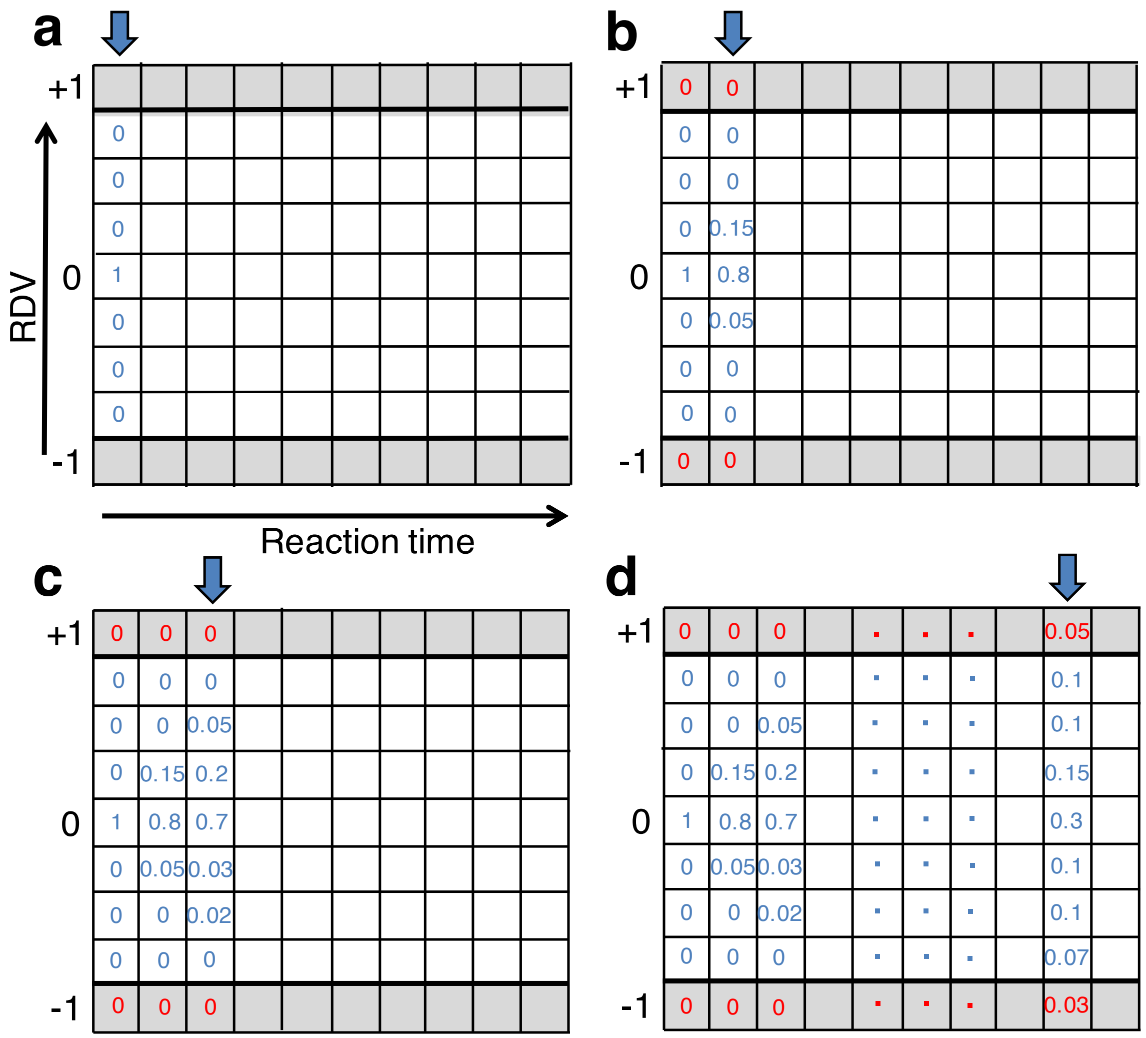

B. Robustness: Additional tests for Experiment 1

In order to better understand the ability of the aDDM to explain the data from Experiment 1, and to better understand the shortcomings of the model, we carried out two additional tests.

Additional test 1

This test was designed to investigate the assumption that perceptual signals are integrated linearly in the aDDM. To do this, we fitted a variation of the aDDM with an additional free parameter α and allowed for the integration of non-linear perceptual signals of the form r_{left}^{α} and r_{right}^{α}.

Let RDV_t denote the relative decision value at time t within the course of a single decision. At every time step Δ_t, its change is given by $\mu\ \Delta_t + \varepsilon_t$, where ε_t is i.i.d. zero mean white Gaussian noise with standard deviation σ, and μ is the deterministic change in the RDV over the time step. $\mu = 0$ until the first fixation to one of the two stimuli occurs, as well as during non-stimuli fixations and inter-fixation transitions, while $\mu = d(r_{left}^{\alpha} - \theta r_{right}^{\alpha})$ during fixations to the left option, and $\mu = d(\theta r_{left}^{\alpha} - r_{right}^{\alpha})$ during fixations to the right option. d is a positive constant that controls the speed of integration, θ is a parameter between 0 and 1 that measures the size of the attentional bias, r_{left} and r_{right} are the relative proximities of the left and right items shown in the trial, and α is the exponent for the relative proximities values. **Figure S3** shows how different values of α affect the transformation from an item's angular distance to the target to its relative proximity value, which is used in the model fitting.

We used the same grid search and MLE procedure described above and in the main text to find the optimal parameters for alternative model 1. We started with a search over the grid obtained from the cross product of $d = \{0.003, 0.004, 0.005\}$, $\sigma = \{0.06, 0.07, 0.08\}$, $\theta = \{0.15, 0.25, 0.35\}$ and $\alpha = \{0, 0.5, 1\}$. We then zoomed into the optimal parameters at each step, making the grid finer, until reaching a stopping criterion (when the improvement in the likelihood of the proposed solution was less than 1%). The resulting solution obtained through this process was $d = 0.0042$, $\sigma = 0.063$, $\theta = 0.37$ and $\alpha = 0.93$. The similarity between this result and the one obtained for the simple aDDM ($d = 0.0041$, $\sigma = 0.063$, $\theta = 0.36$ and $\alpha = 1$) suggests that the simpler model, with $\alpha = 1$, can capture many of the patterns observed in the behavioral data without the need for non-linear perceptual signals. **Figure S4** shows a comparison of some of these behavioral patterns in the data and in the simulations generated for alternative model 1.

Additional test 2

This test was designed to investigate if the best fitting parameters of the aDDM change within the course of a block of trials, as the memory of the target orientation dissipates.

To do this, we fitted the model separately for trials occurring immediately after target display (early trials) and trials occurring 4 trials after target display (late trials).

Alternative model 2 is the same as the aDDM we describe in the main text. Let RDV_t denote the relative decision value at time t within the course of a single decision. At every time step Δ_t, its change is given by $\mu \Delta_t + \varepsilon_t$, where ε_t is i.i.d. zero mean white Gaussian noise with standard deviation σ, and μ is the deterministic change in the RDV over the time step. $\mu = 0$ until the first fixation to one of the two stimuli occurs, as well as during non-stimuli fixations and inter-fixation transitions, while $\mu = d(r_{left} - \theta r_{right})$ during fixations to the left option, and $\mu = d(\theta r_{left} - r_{right})$ during fixations to the right option. d is a positive constant that controls the speed of integration, θ is a parameter between 0 and 1 that measures the size of the attentional bias, and r_{left} and r_{right} are the relative proximities of the left and right items shown in the trial.

We fit the model separately for two sets of trials, first using only trials occurring immediately after target display (early trials), in which the memory of the target is at its most recent for the subjects, then using only trials occurring 4 trials after target display (late trials), in which the memory decay of the target is at its peak. We used the same grid search and MLE procedure described above and in the main text to find the optimal parameters for these two instances of alternative model 2. Using early trials only, the optimal parameters obtained were $d = 0.0042$, $\sigma = 0.063$ and $\theta = 0.36$; using late trials only, the optimal parameters were $d = 0.004$, $\sigma = 0.063$ and $\theta = 0.37$. The similarity between these two results shows that we can use all trials in the same model fitting procedure, as the position of the trial in the timeline of the experiment does not affect the behavioral results in a way that significantly impacts the model fitting results (see also **Figure S5** for a comparison between the behavioral results for early and late trials).

Figure S3. Conversion from an item's angular distance to the target (Δ) to a relative proximity value (r) to be used in the model fitting, for different values of α. The aDDM fitted in the main paper uses $\alpha = 1$, whereas other values of α were tested in alternative model 1, as discussed in these Supplementary Materials.

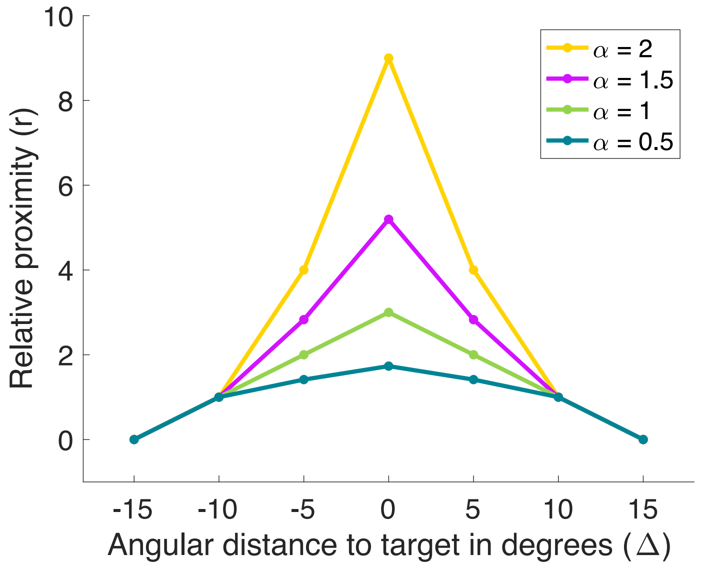

Figure S4. Comparison between data trials and simulations of the non-linear version of the aDDM. (**a**) Psychometric choice curve. (**b**) RT curve depicting mean response times versus trial ease, as measured by the difference in absolute proximity between the correct and incorrect options. (**c**) Mean number of fixations versus trial ease. (**d**) Psychometric choice curves conditioned on the location of the last fixation. (**e**) Probability that left item is chosen as a function of the excess amount of time for which the left item was fixated during the trial. (**f**) Probability that the first-seen item is chosen as a function of the duration of that first fixation. Subject data includes only even-numbered trials. Error bars show 95% confidence intervals for the subject's data pooled across all even trials, and across all simulated trials in the case of the model.

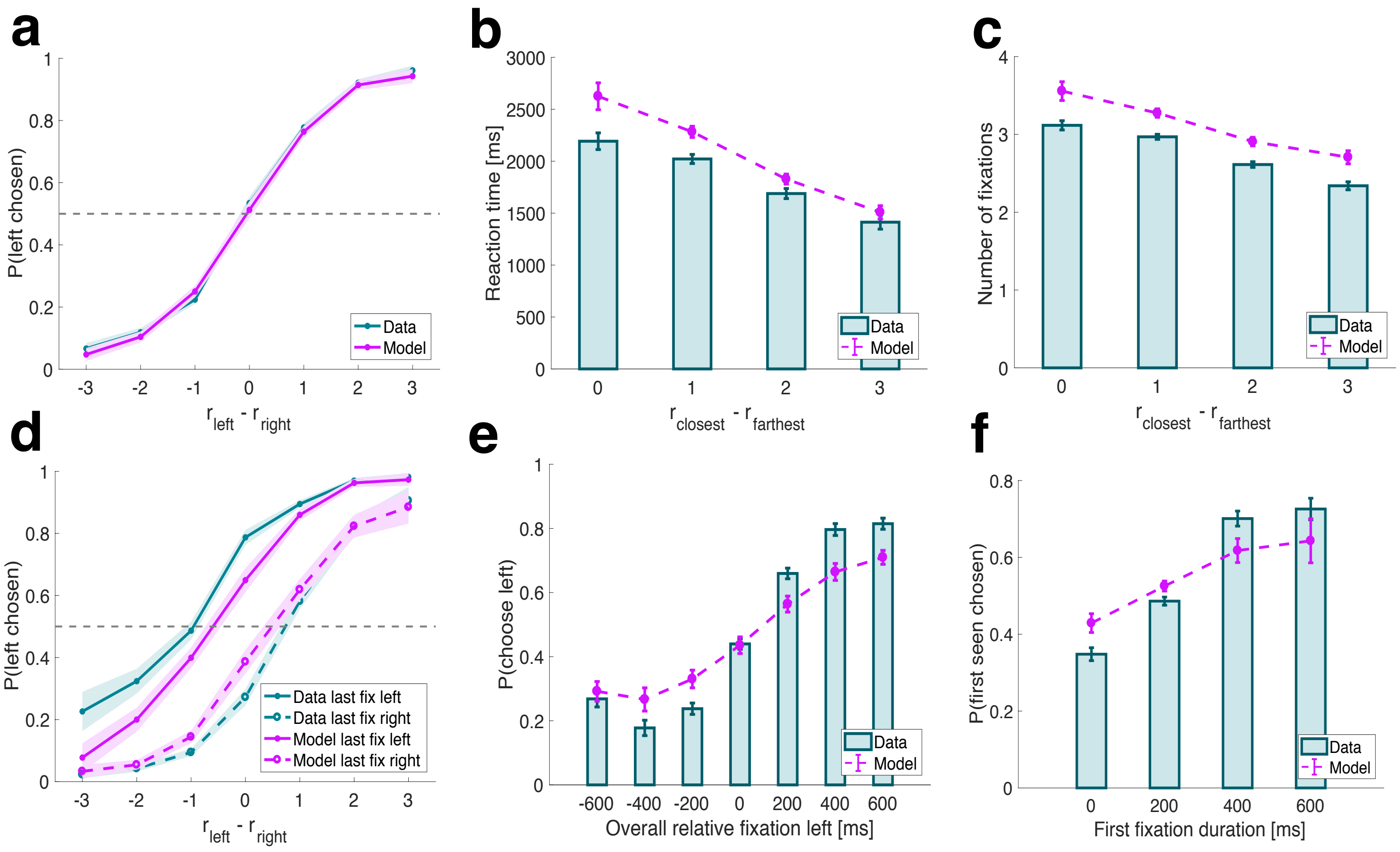

Figure S5. Comparison between trials where the memory of the target is recent vs. old. (**a**) Psychometric choice curve. (**b**) RT curve depicting mean response times versus trial ease, as measured by the difference in absolute proximity between the correct and incorrect options. (**c**) Mean number of fixations versus trial ease. (**d**) Psychometric choice curves conditioned on the location of the last fixation. (**e**) Probability that left item is chosen as a function of the excess amount of time for which the left item was fixated during the trial. (**f**) Probability that the first-seen item is chosen as a function of the duration of that first fixation. Early trials are those occurring immediately after target display, while late trials are those occurring 4 trials after target display. Error bars show 95% confidence intervals for the data pooled across all subjects.

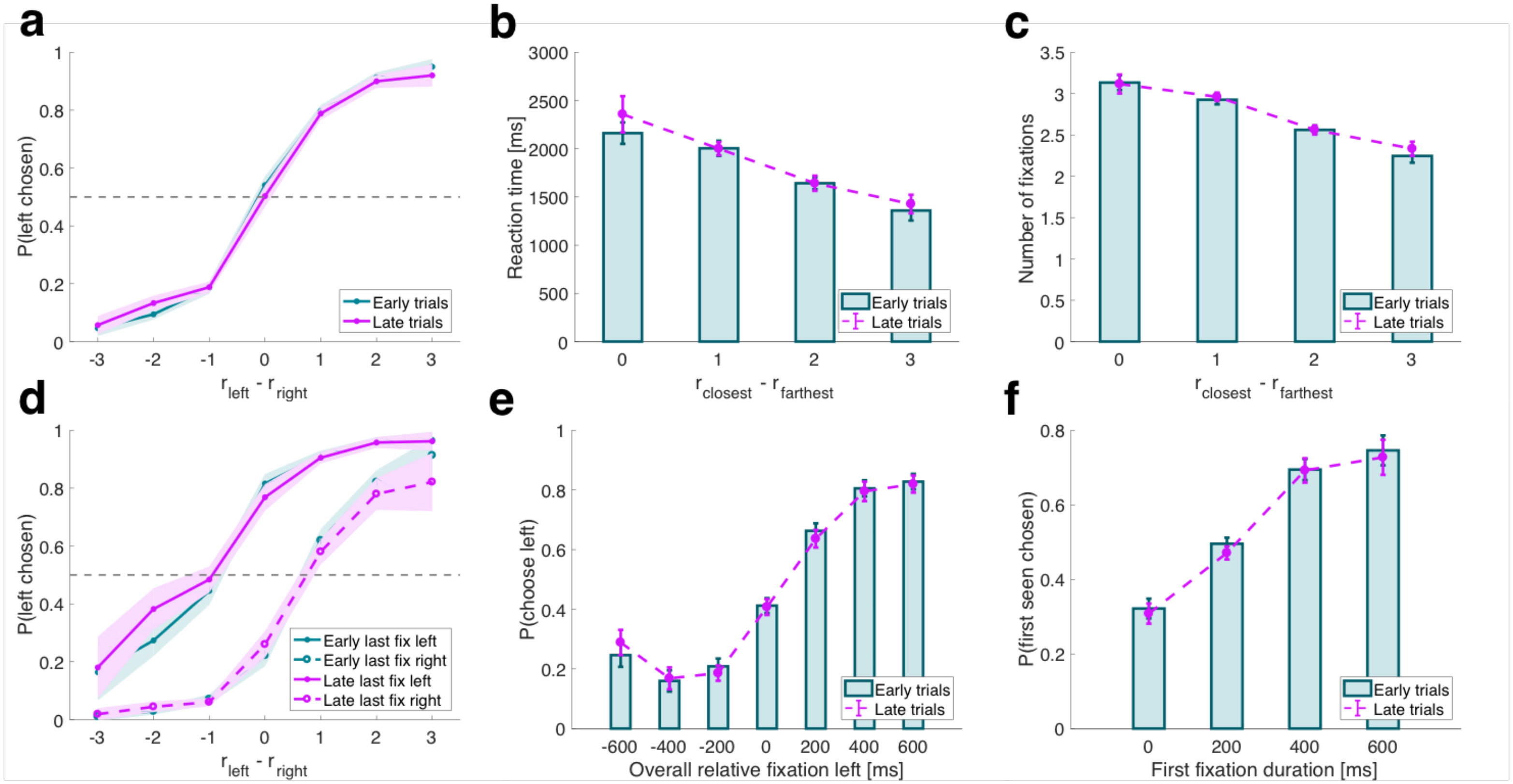

C. Individual subject fits for Experiment 1

Figure S6. Histograms of the aDDM free parameters fitted per subject (N = 25).

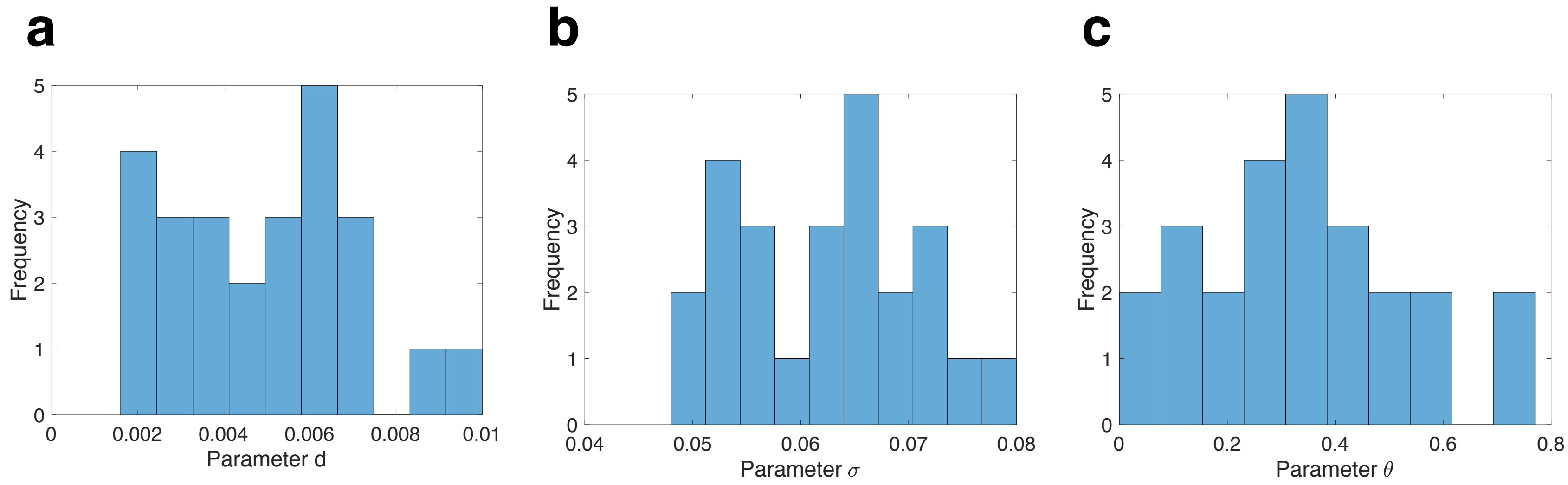

Figure S7. Results for subject 01. Fitted aDDM parameters: $d = 0.007, \sigma = 0.063, \theta = 0.16$. (**a**) Psychometric choice curve. (**b**) RT curve depicting mean response times versus trial ease, as measured by the difference in absolute proximity between the correct and incorrect options. (**c**) Mean number of fixations versus trial ease. (**d**) Psychometric choice curves conditioned on the location of the last fixation. (**e**) Probability that left item is chosen as a function of the excess amount of time for which the left item was fixated during the trial. (**f**) Probability that the first-seen item is chosen as a function of the duration of that first fixation. Subject data includes only even-numbered trials. Error bars show 95% confidence intervals for the subject's data pooled across all even trials, and across all simulated trials in the case of the model.

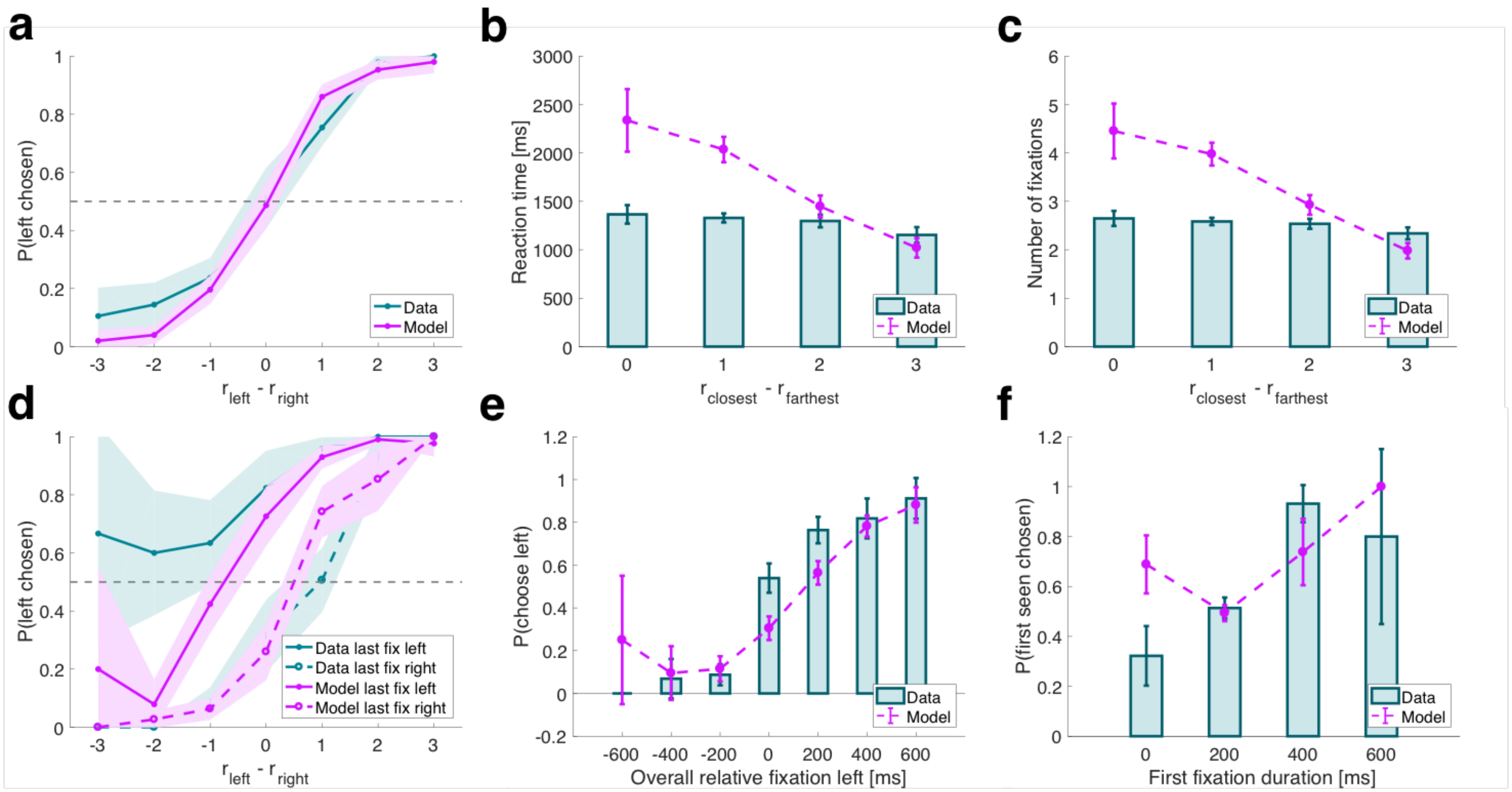

Figure S8. Results for subject 02. Fitted aDDM parameters: $d = 0.0065, \sigma = 0.063, \theta = 0.38$. (**a**) Psychometric choice curve. (**b**) RT curve depicting mean response times versus trial ease, as measured by the difference in absolute proximity between the correct and incorrect options. (**c**) Mean number of fixations versus trial ease. (**d**) Psychometric choice curves conditioned on the location of the last fixation. (**e**) Probability that left item is chosen as a function of the excess amount of time for which the left item was fixated during the trial. (**f**) Probability that the first-seen item is chosen as a function of the duration of that first fixation. Subject data includes only even-numbered trials. Error bars show 95% confidence intervals for the subject's data pooled across all even trials, and across all simulated trials in the case of the model.

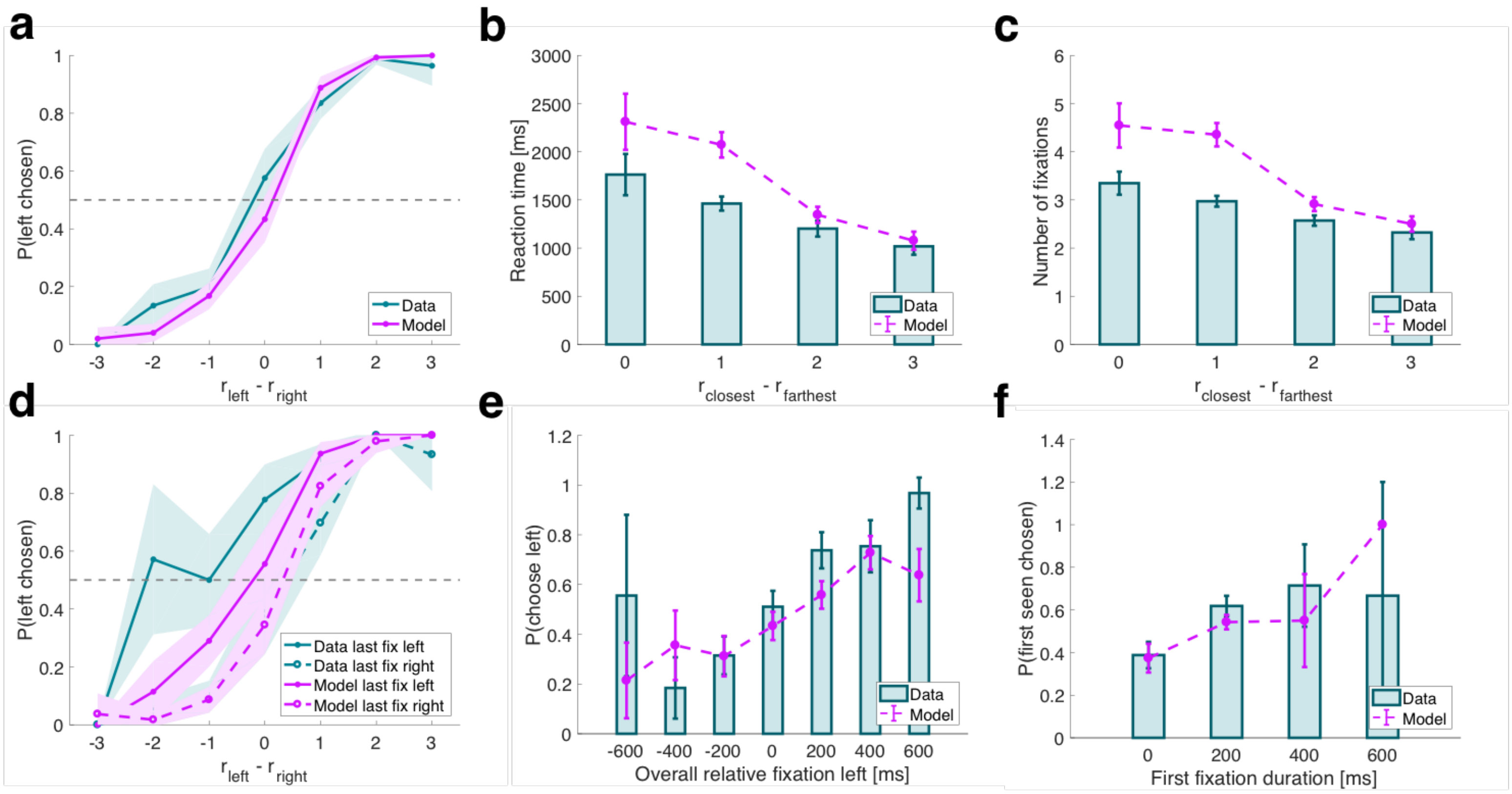

Figure S9. Results for subject 03. Fitted aDDM parameters: $d = 0.0035, \sigma = 0.055, \theta = 0.27$. (**a**) Psychometric choice curve. (**b**) RT curve depicting mean response times versus trial ease, as measured by the difference in absolute proximity between the correct and incorrect options. (**c**) Mean number of fixations versus trial ease. (**d**) Psychometric choice curves conditioned on the location of the last fixation. (**e**) Probability that left item is chosen as a function of the excess amount of time for which the left item was fixated during the trial. (**f**) Probability that the first-seen item is chosen as a function of the duration of that first fixation. Subject data includes only even-numbered trials. Error bars show 95% confidence intervals for the subject's data pooled across all even trials, and across all simulated trials in the case of the model.

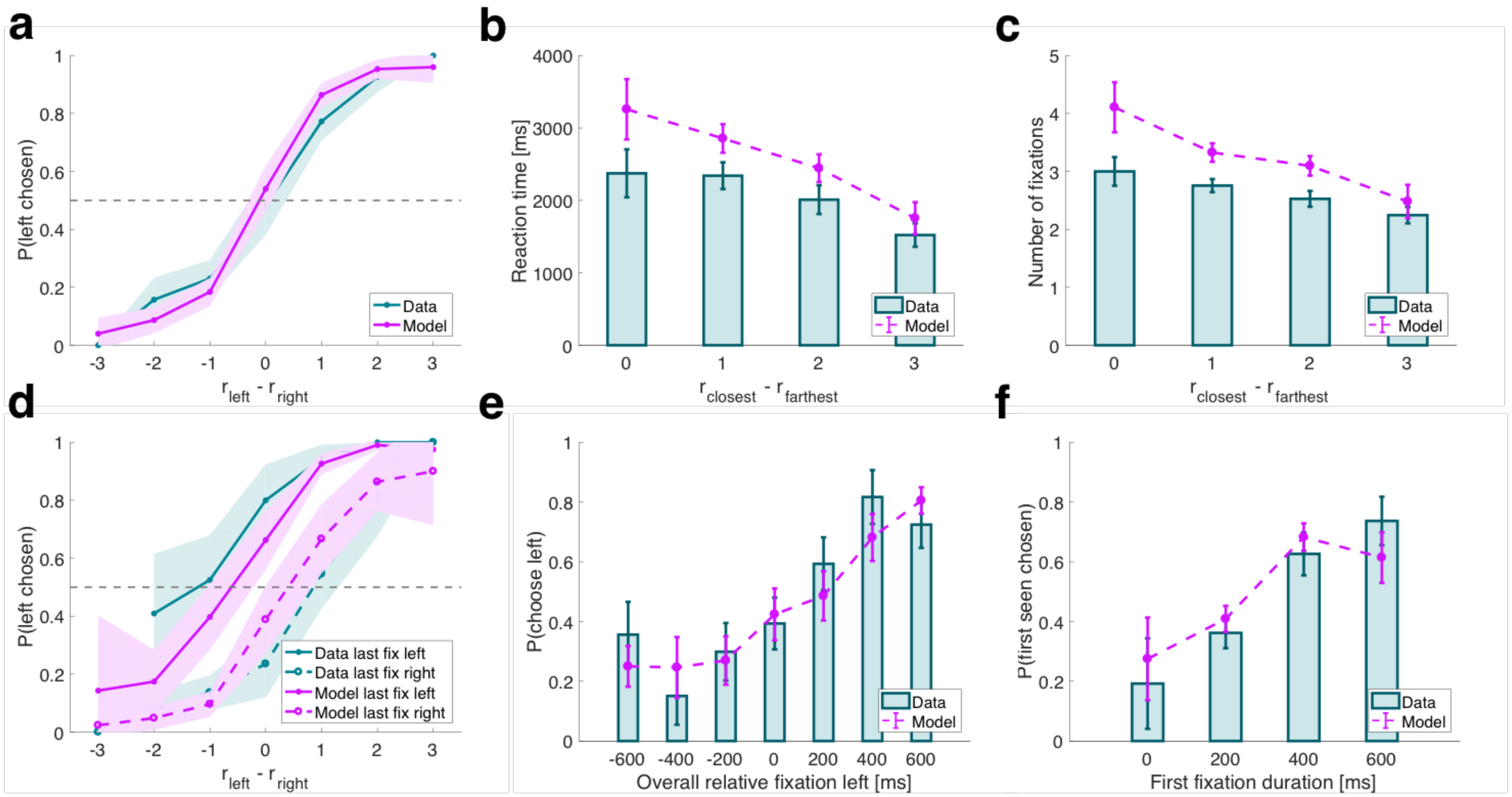

Figure S10. Results for subject 04. Fitted aDDM parameters: $d = 0.0063, \sigma = 0.054, \theta = 0.53$. (**a**) Psychometric choice curve. (**b**) RT curve depicting mean response times versus trial ease, as measured by the difference in absolute proximity between the correct and incorrect options. (**c**) Mean number of fixations versus trial ease. (**d**) Psychometric choice curves conditioned on the location of the last fixation. (**e**) Probability that left item is chosen as a function of the excess amount of time for which the left item was fixated during the trial. (**f**) Probability that the first-seen item is chosen as a function of the duration of that first fixation. Subject data includes only even-numbered trials. Error bars show 95% confidence intervals for the subject's data pooled across all even trials, and across all simulated trials in the case of the model.

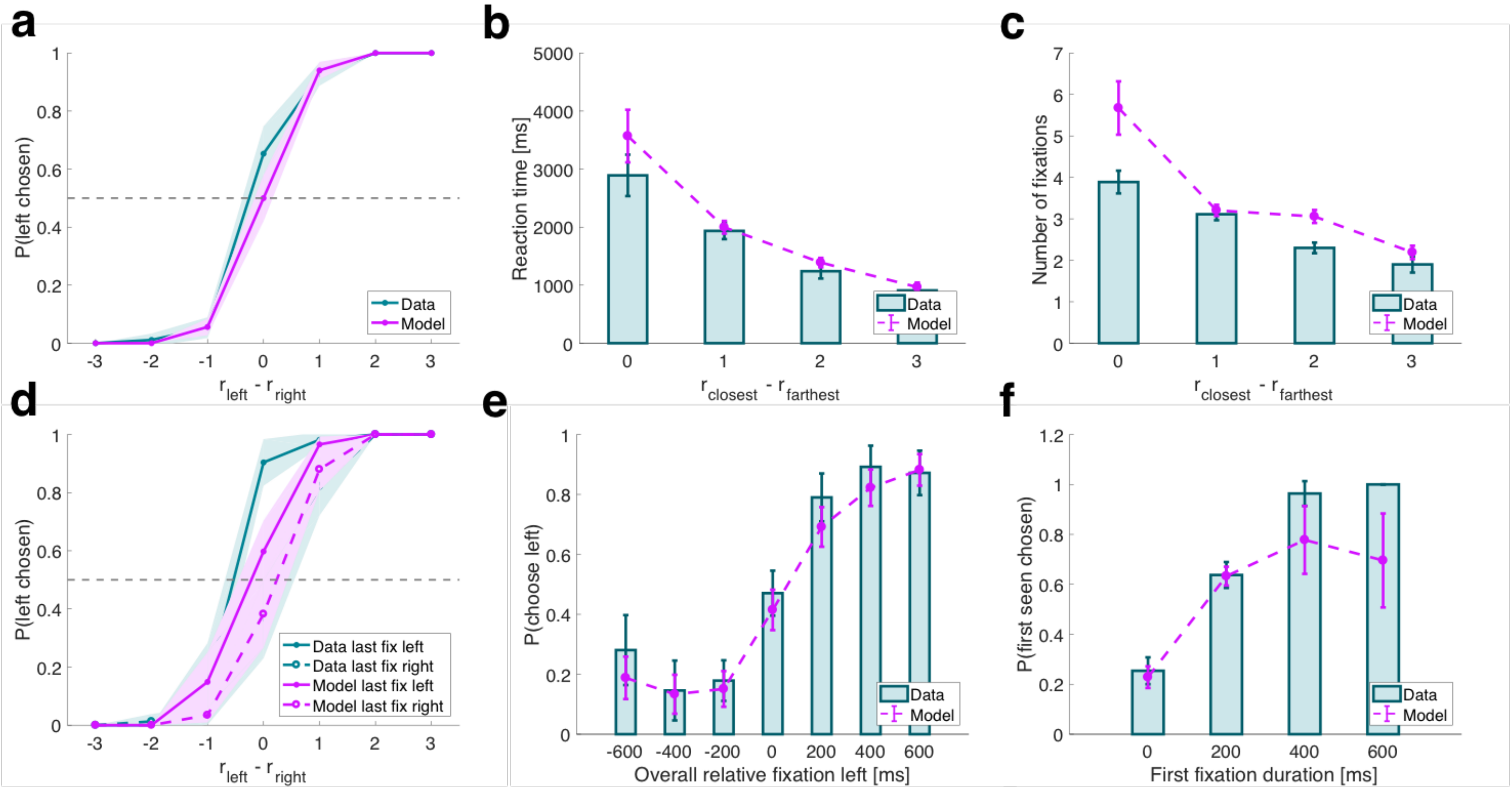

Figure S11. Results for subject 05. Fitted aDDM parameters: $d = 0.0055, \sigma = 0.065, \theta = 0.12$. (**a**) Psychometric choice curve. (**b**) RT curve depicting mean response times versus trial ease, as measured by the difference in absolute proximity between the correct and incorrect options. (**c**) Mean number of fixations versus trial ease. (**d**) Psychometric choice curves conditioned on the location of the last fixation. (**e**) Probability that left item is chosen as a function of the excess amount of time for which the left item was fixated during the trial. (**f**) Probability that the first-seen item is chosen as a function of the duration of that first fixation. Subject data includes only even-numbered trials. Error bars show 95% confidence intervals for the subject's data pooled across all even trials, and across all simulated trials in the case of the model.

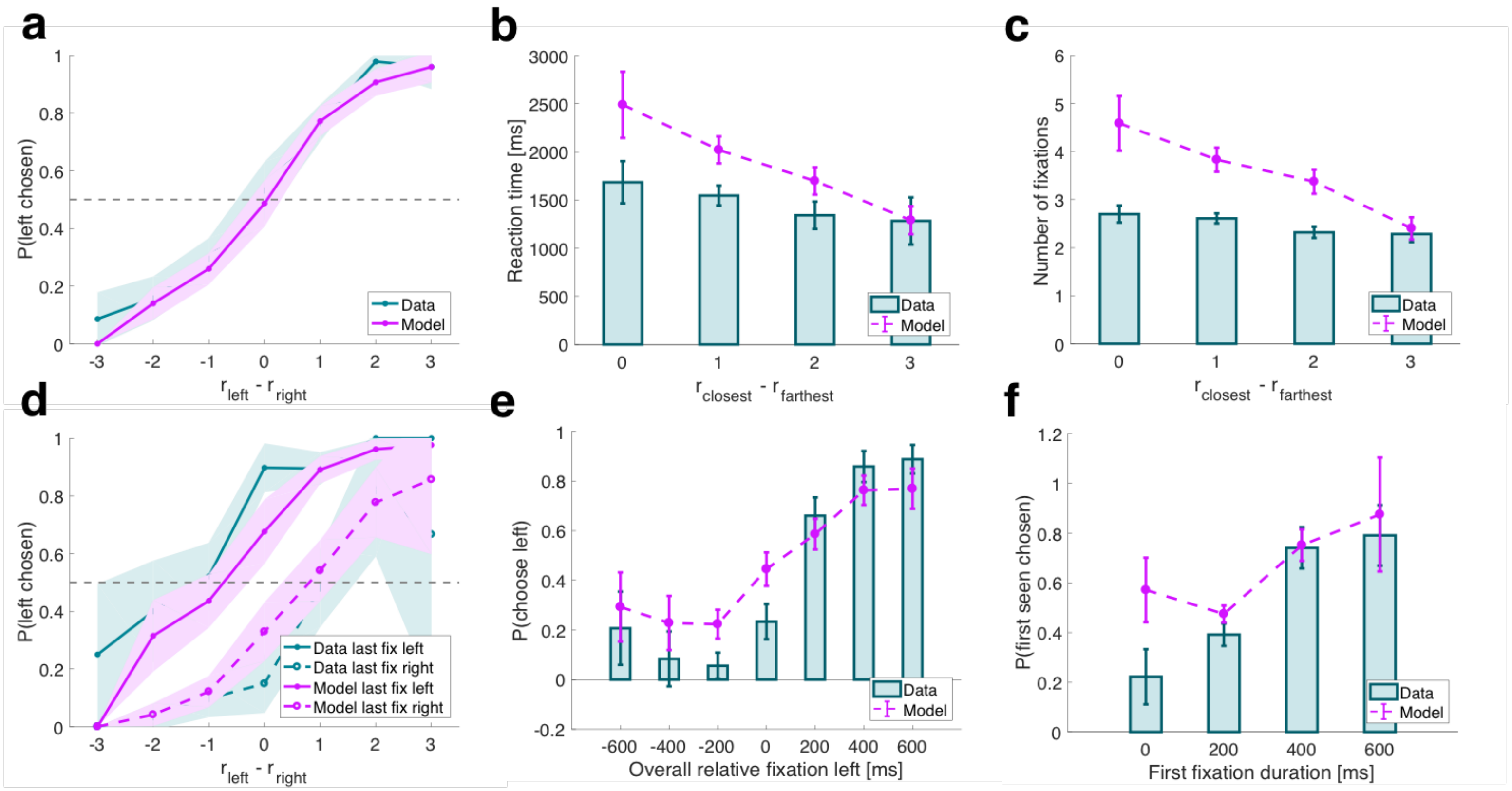

Figure S12. Results for subject 06. Fitted aDDM parameters: $d = 0.0052, \sigma = 0.064, \theta = 0.18$. (**a**) Psychometric choice curve. (**b**) RT curve depicting mean response times versus trial ease, as measured by the difference in absolute proximity between the correct and incorrect options. (**c**) Mean number of fixations versus trial ease. (**d**) Psychometric choice curves conditioned on the location of the last fixation. (**e**) Probability that left item is chosen as a function of the excess amount of time for which the left item was fixated during the trial. (**f**) Probability that the first-seen item is chosen as a function of the duration of that first fixation. Subject data includes only even-numbered trials. Error bars show 95% confidence intervals for the subject's data pooled across all even trials, and across all simulated trials in the case of the model.

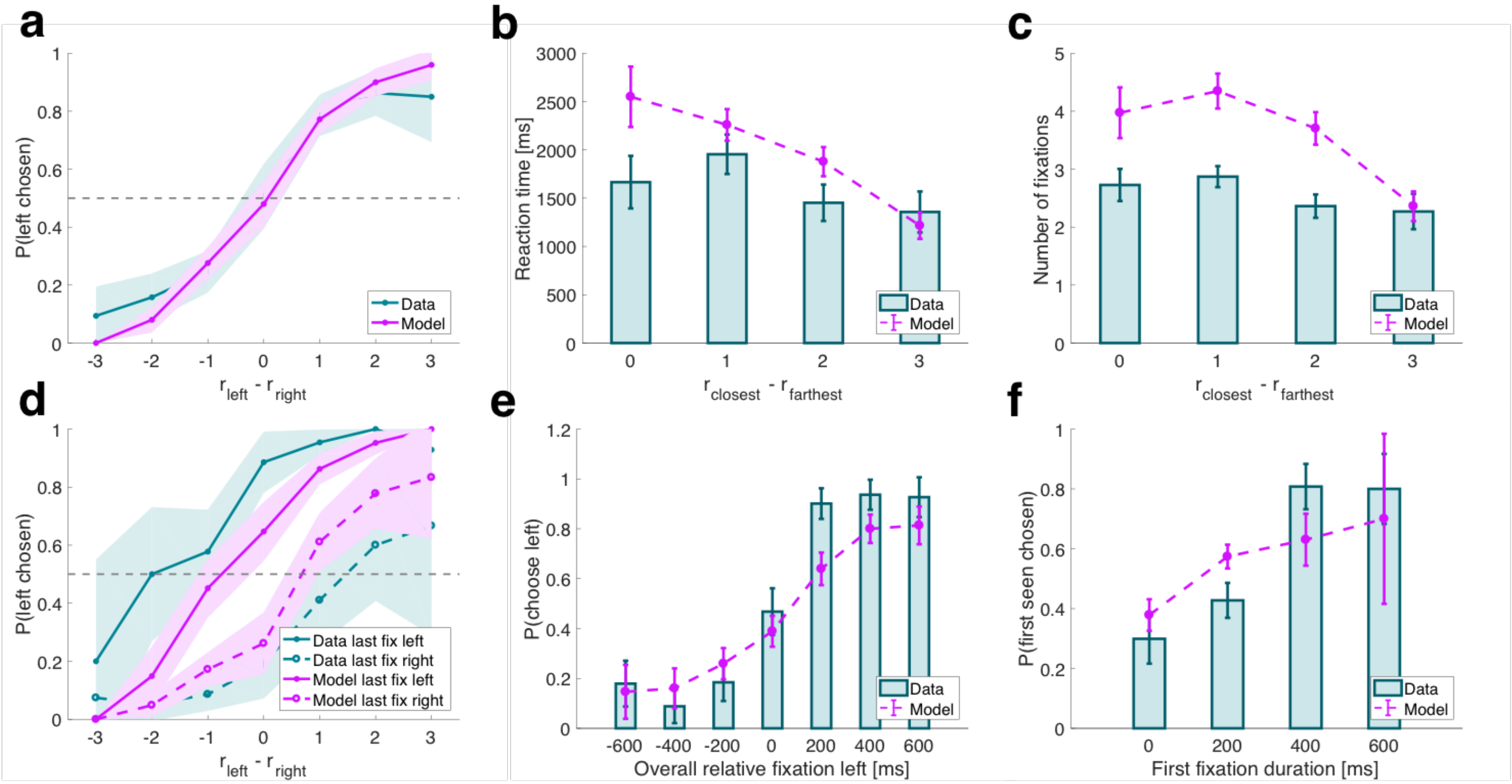

Figure S13. Results for subject 07. Fitted aDDM parameters: $d = 0.002, \sigma = 0.05, \theta = 0.51$. (**a**) Psychometric choice curve. (**b**) RT curve depicting mean response times versus trial ease, as measured by the difference in absolute proximity between the correct and incorrect options. (**c**) Mean number of fixations versus trial ease. (**d**) Psychometric choice curves conditioned on the location of the last fixation. (**e**) Probability that left item is chosen as a function of the excess amount of time for which the left item was fixated during the trial. (**f**) Probability that the first-seen item is chosen as a function of the duration of that first fixation. Subject data includes only even-numbered trials. Error bars show 95% confidence intervals for the subject's data pooled across all even trials, and across all simulated trials in the case of the model.

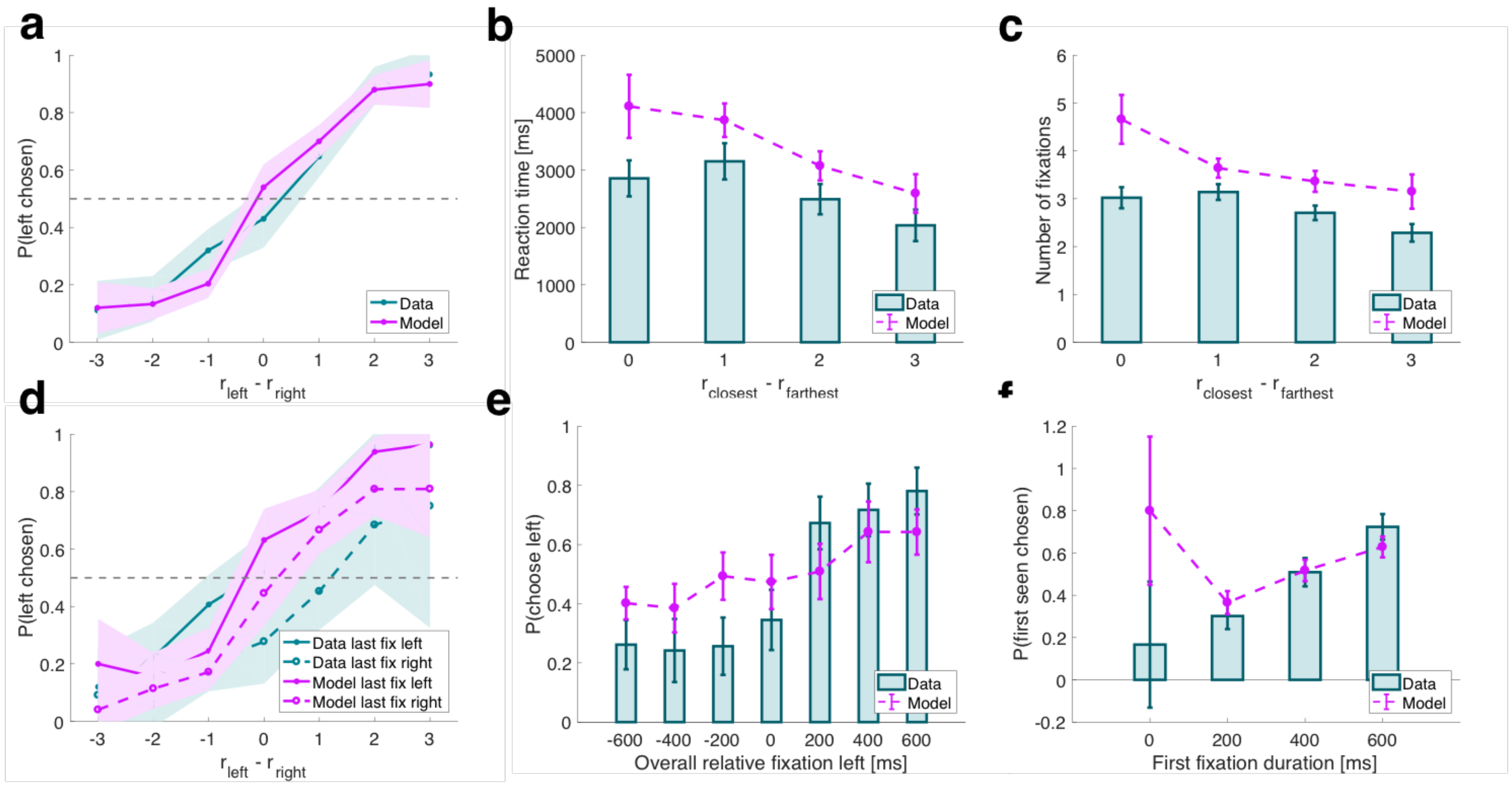

Figure S14. Results for subject 08. Fitted aDDM parameters: $d = 0.0032, \sigma = 0.062, \theta = 0.15$. (**a**) Psychometric choice curve. (**b**) RT curve depicting mean response times versus trial ease, as measured by the difference in absolute proximity between the correct and incorrect options. (**c**) Mean number of fixations versus trial ease. (**d**) Psychometric choice curves conditioned on the location of the last fixation. (**e**) Probability that left item is chosen as a function of the excess amount of time for which the left item was fixated during the trial. (**f**) Probability that the first-seen item is chosen as a function of the duration of that first fixation. Subject data includes only even-numbered trials. Error bars show 95% confidence intervals for the subject's data pooled across all even trials, and across all simulated trials in the case of the model.

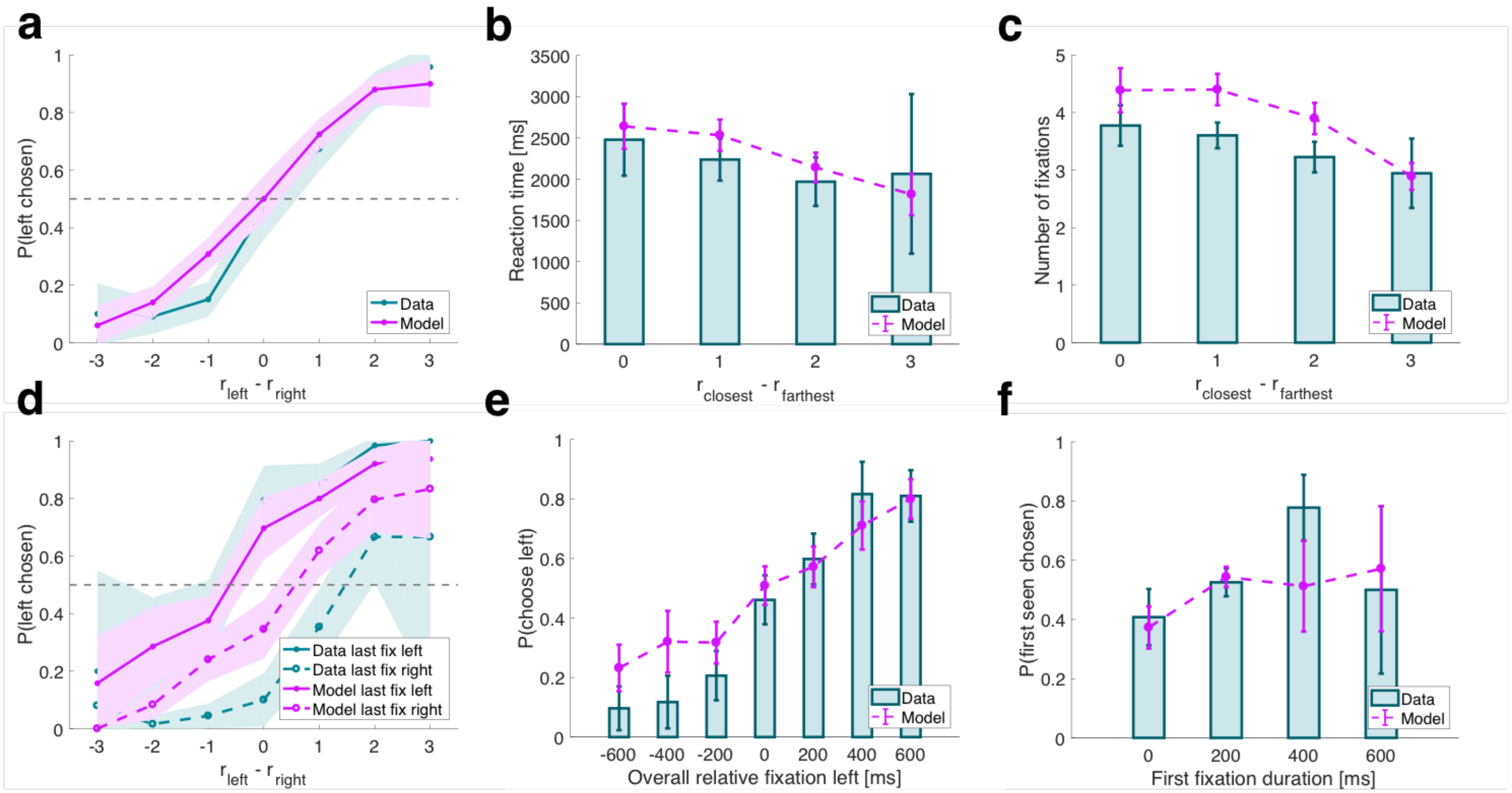

Figure S15. Results for subject 09. Fitted aDDM parameters: $d = 0.006, \sigma = 0.065, \theta = 0.72$. (**a**) Psychometric choice curve. (**b**) RT curve depicting mean response times versus trial ease, as measured by the difference in absolute proximity between the correct and incorrect options. (**c**) Mean number of fixations versus trial ease. (**d**) Psychometric choice curves conditioned on the location of the last fixation. (**e**) Probability that left item is chosen as a function of the excess amount of time for which the left item was fixated during the trial. (**f**) Probability that the first-seen item is chosen as a function of the duration of that first fixation. Subject data includes only even-numbered trials. Error bars show 95% confidence intervals for the subject's data pooled across all even trials, and across all simulated trials in the case of the model.

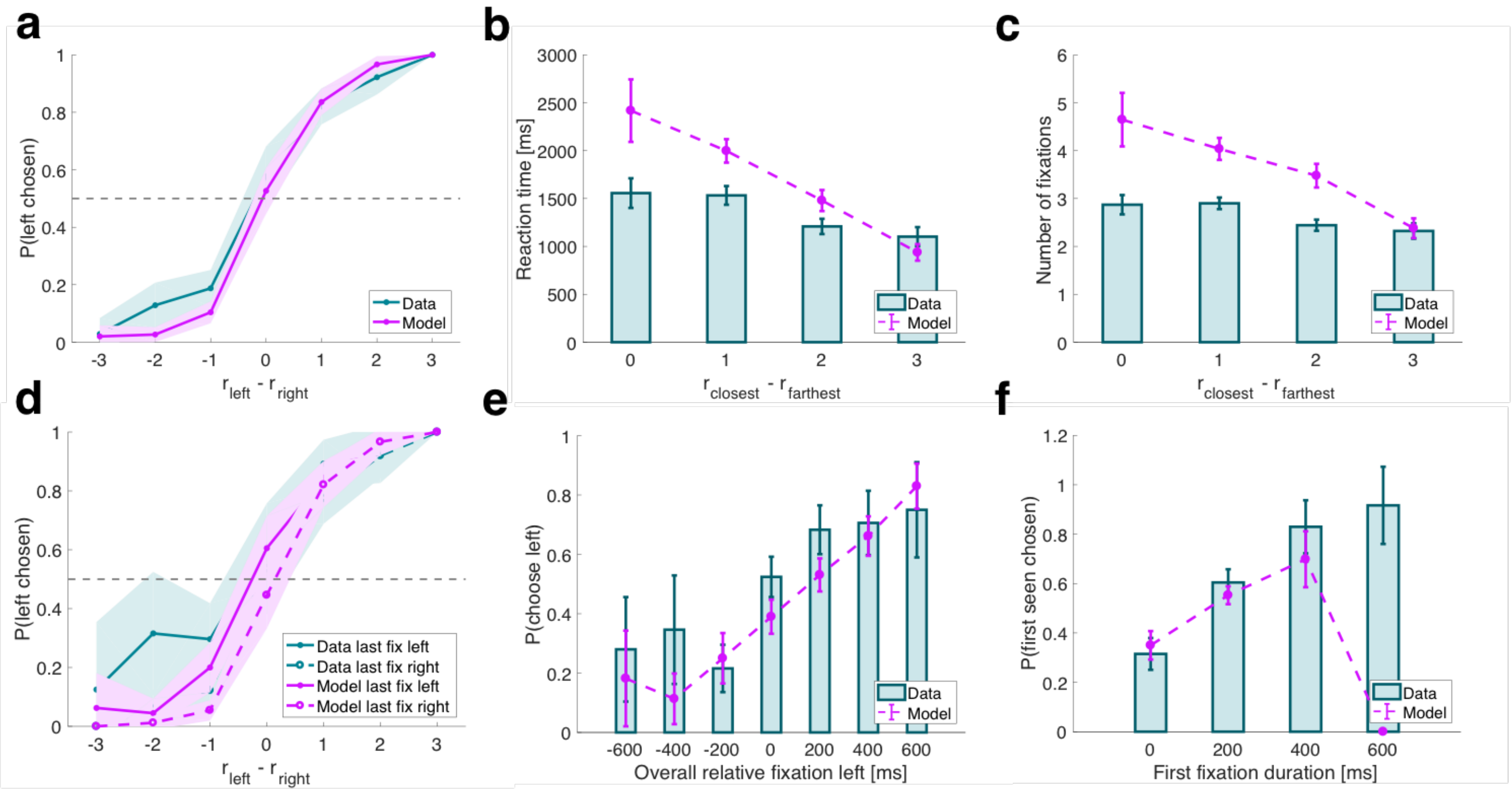

Figure S16. Results for subject 10. Fitted aDDM parameters: $d = 0.0072, \sigma = 0.067, \theta = 0.58$. (**a**) Psychometric choice curve. (**b**) RT curve depicting mean response times versus trial ease, as measured by the difference in absolute proximity between the correct and incorrect options. (**c**) Mean number of fixations versus trial ease. (**d**) Psychometric choice curves conditioned on the location of the last fixation. (**e**) Probability that left item is chosen as a function of the excess amount of time for which the left item was fixated during the trial. (**f**) Probability that the first-seen item is chosen as a function of the duration of that first fixation. Subject data includes only even-numbered trials. Error bars show 95% confidence intervals for the subject's data pooled across all even trials, and across all simulated trials in the case of the model.

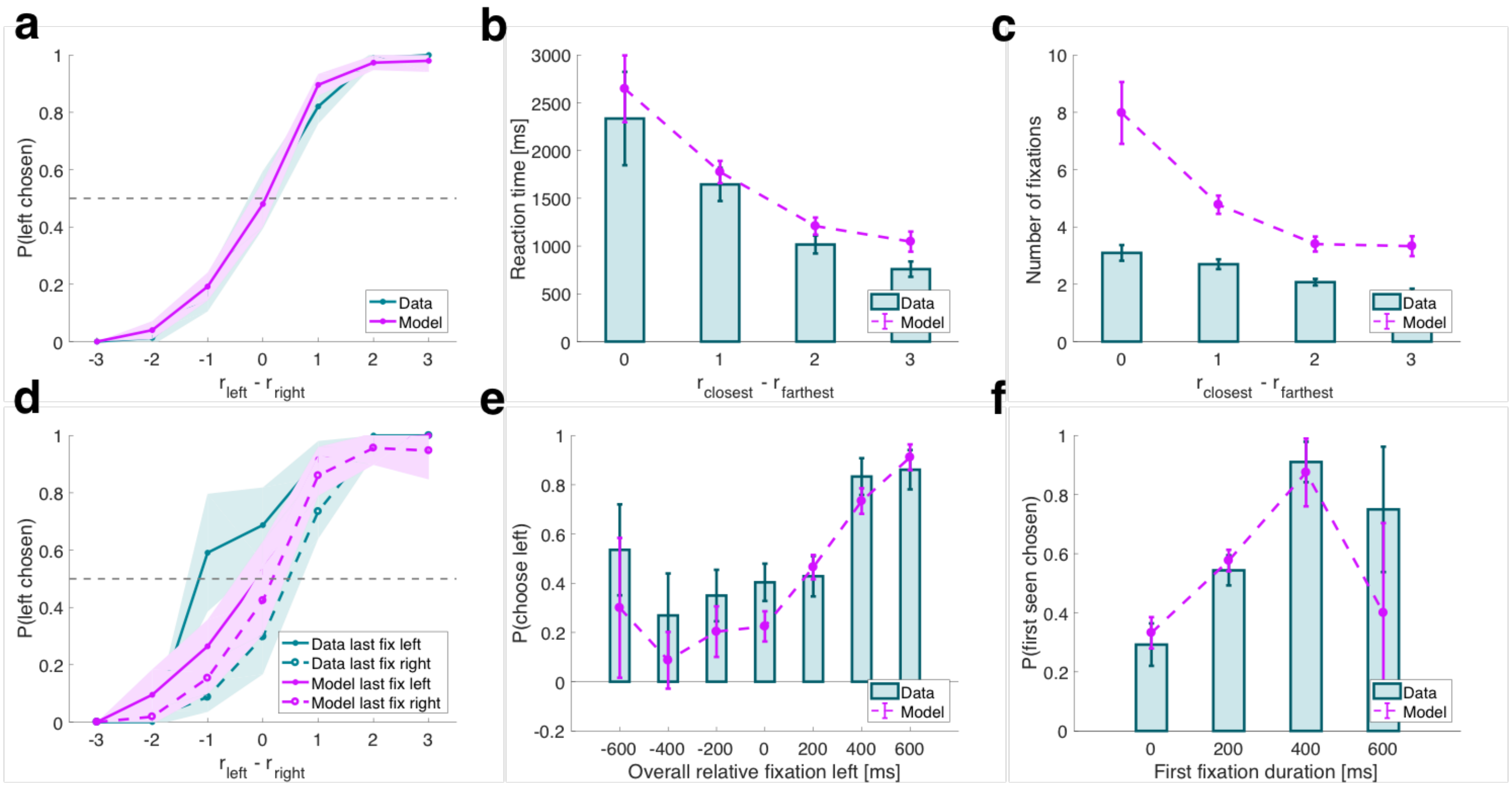

Figure S17. Results for subject 11. Fitted aDDM parameters: $d = 0.0074, \sigma = 0.08, \theta = 0.04$. (**a**) Psychometric choice curve. (**b**) RT curve depicting mean response times versus trial ease, as measured by the difference in absolute proximity between the correct and incorrect options. (**c**) Mean number of fixations versus trial ease. (**d**) Psychometric choice curves conditioned on the location of the last fixation. (**e**) Probability that left item is chosen as a function of the excess amount of time for which the left item was fixated during the trial. (**f**) Probability that the first-seen item is chosen as a function of the duration of that first fixation. Subject data includes only even-numbered trials. Error bars show 95% confidence intervals for the subject's data pooled across all even trials, and across all simulated trials in the case of the model.

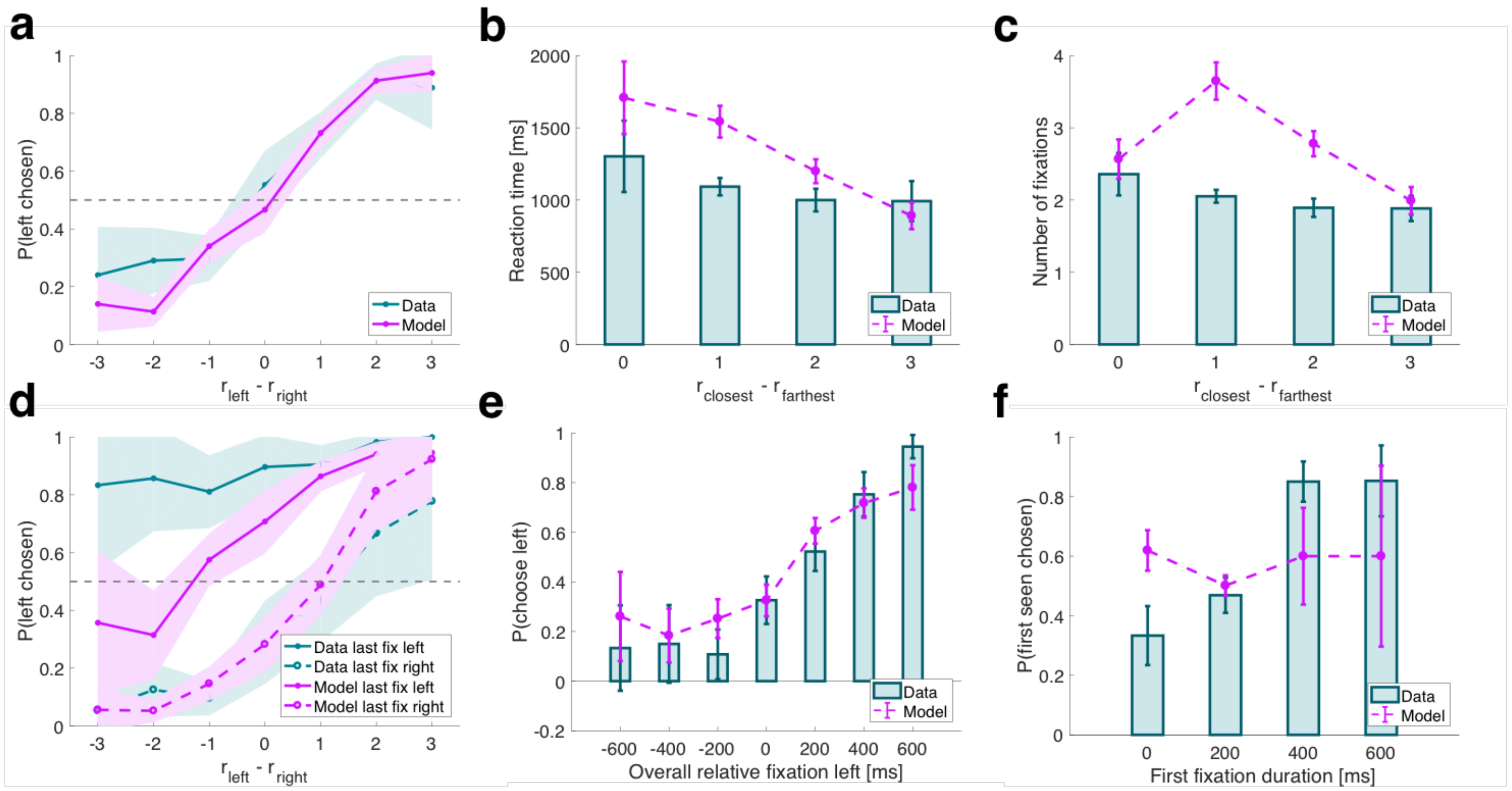

Figure S18. Results for subject 12. Fitted aDDM parameters: $d = 0.0055, \sigma = 0.065, \theta = 0.6$. (**a**) Psychometric choice curve. (**b**) RT curve depicting mean response times versus trial ease, as measured by the difference in absolute proximity between the correct and incorrect options. (**c**) Mean number of fixations versus trial ease. (**d**) Psychometric choice curves conditioned on the location of the last fixation. (**e**) Probability that left item is chosen as a function of the excess amount of time for which the left item was fixated during the trial. (**f**) Probability that the first-seen item is chosen as a function of the duration of that first fixation. Subject data includes only even-numbered trials. Error bars show 95% confidence intervals for the subject's data pooled across all even trials, and across all simulated trials in the case of the model.

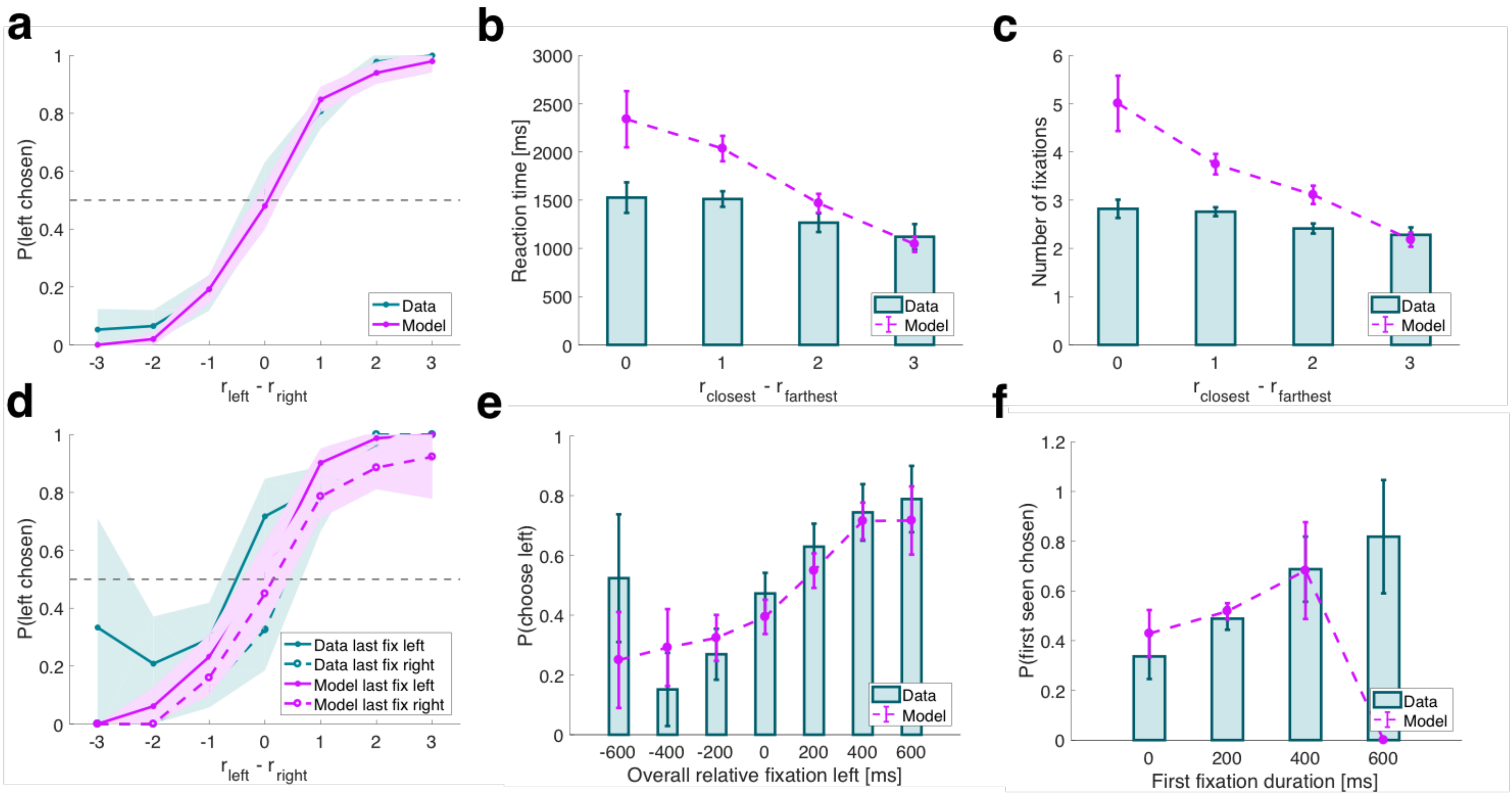

Figure S19. Results for subject 13. Fitted aDDM parameters: $d = 0.0049, \sigma = 0.058, \theta = 0.4$. (**a**) Psychometric choice curve. (**b**) RT curve depicting mean response times versus trial ease, as measured by the difference in absolute proximity between the correct and incorrect options. (**c**) Mean number of fixations versus trial ease. (**d**) Psychometric choice curves conditioned on the location of the last fixation. (**e**) Probability that left item is chosen as a function of the excess amount of time for which the left item was fixated during the trial. (**f**) Probability that the first-seen item is chosen as a function of the duration of that first fixation. Subject data includes only even-numbered trials. Error bars show 95% confidence intervals for the subject's data pooled across all even trials, and across all simulated trials in the case of the model.

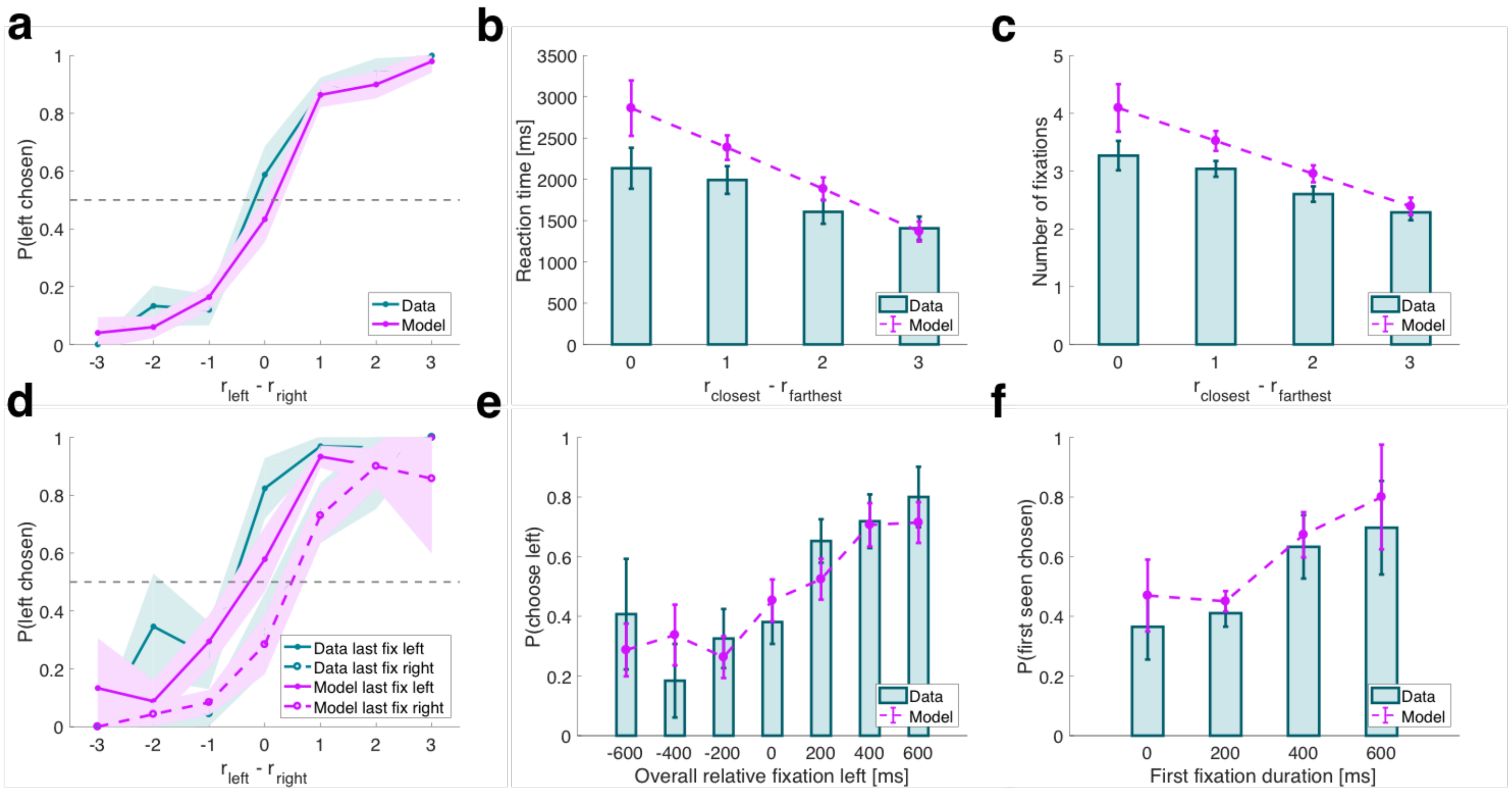

Figure S20. Results for subject 14. Fitted aDDM parameters: $d = 0.0032, \sigma = 0.054, \theta = 0.35$. (**a**) Psychometric choice curve. (**b**) RT curve depicting mean response times versus trial ease, as measured by the difference in absolute proximity between the correct and incorrect options. (**c**) Mean number of fixations versus trial ease. (**d**) Psychometric choice curves conditioned on the location of the last fixation. (**e**) Probability that left item is chosen as a function of the excess amount of time for which the left item was fixated during the trial. (**f**) Probability that the first-seen item is chosen as a function of the duration of that first fixation. Subject data includes only even-numbered trials. Error bars show 95% confidence intervals for the subject's data pooled across all even trials, and across all simulated trials in the case of the model.

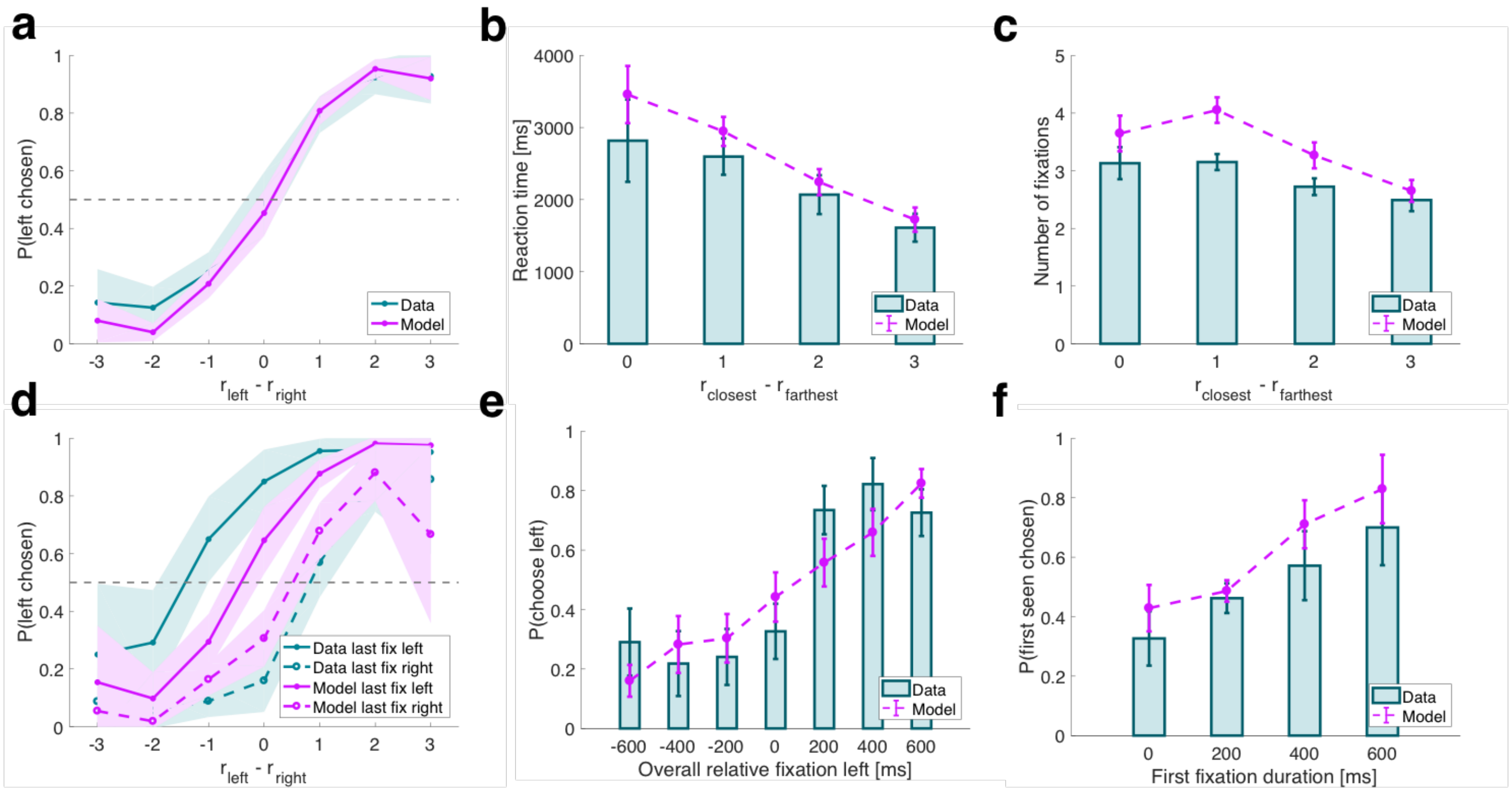

Figure S21. Results for subject 15. Fitted aDDM parameters: $d = 0.0023, \sigma = 0.056, \theta = 0.32$. (**a**) Psychometric choice curve. (**b**) RT curve depicting mean response times versus trial ease, as measured by the difference in absolute proximity between the correct and incorrect options. (**c**) Mean number of fixations versus trial ease. (**d**) Psychometric choice curves conditioned on the location of the last fixation. (**e**) Probability that left item is chosen as a function of the excess amount of time for which the left item was fixated during the trial. (**f**) Probability that the first-seen item is chosen as a function of the duration of that first fixation. Subject data includes only even-numbered trials. Error bars show 95% confidence intervals for the subject's data pooled across all even trials, and across all simulated trials in the case of the model.

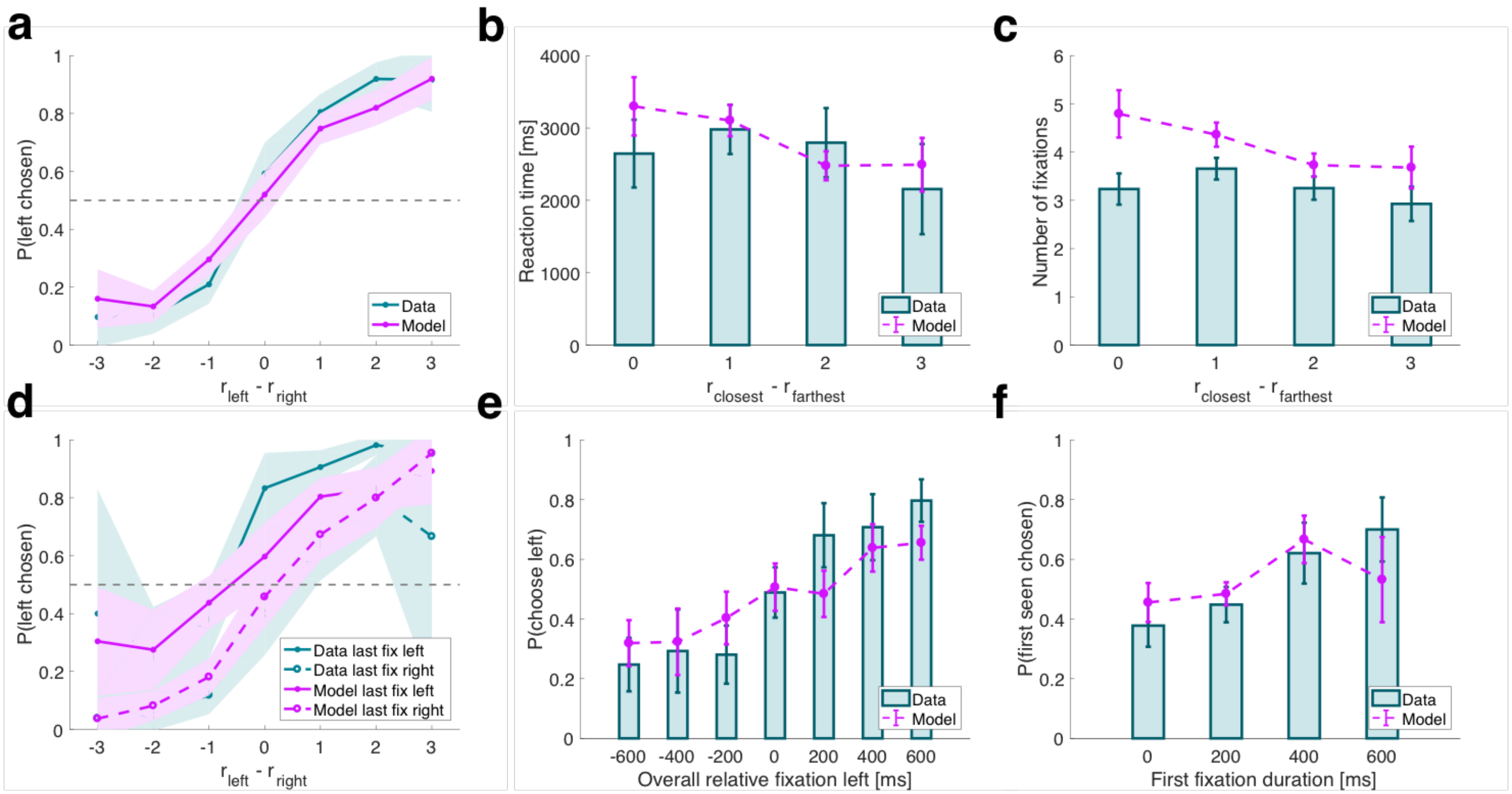

Figure S22. Results for subject 16. Fitted aDDM parameters: $d = 0.006, \sigma = 0.073, \theta = 0.3$. (**a**) Psychometric choice curve. (**b**) RT curve depicting mean response times versus trial ease, as measured by the difference in absolute proximity between the correct and incorrect options. (**c**) Mean number of fixations versus trial ease. (**d**) Psychometric choice curves conditioned on the location of the last fixation. (**e**) Probability that left item is chosen as a function of the excess amount of time for which the left item was fixated during the trial. (**f**) Probability that the first-seen item is chosen as a function of the duration of that first fixation. Subject data includes only even-numbered trials. Error bars show 95% confidence intervals for the subject's data pooled across all even trials, and across all simulated trials in the case of the model.

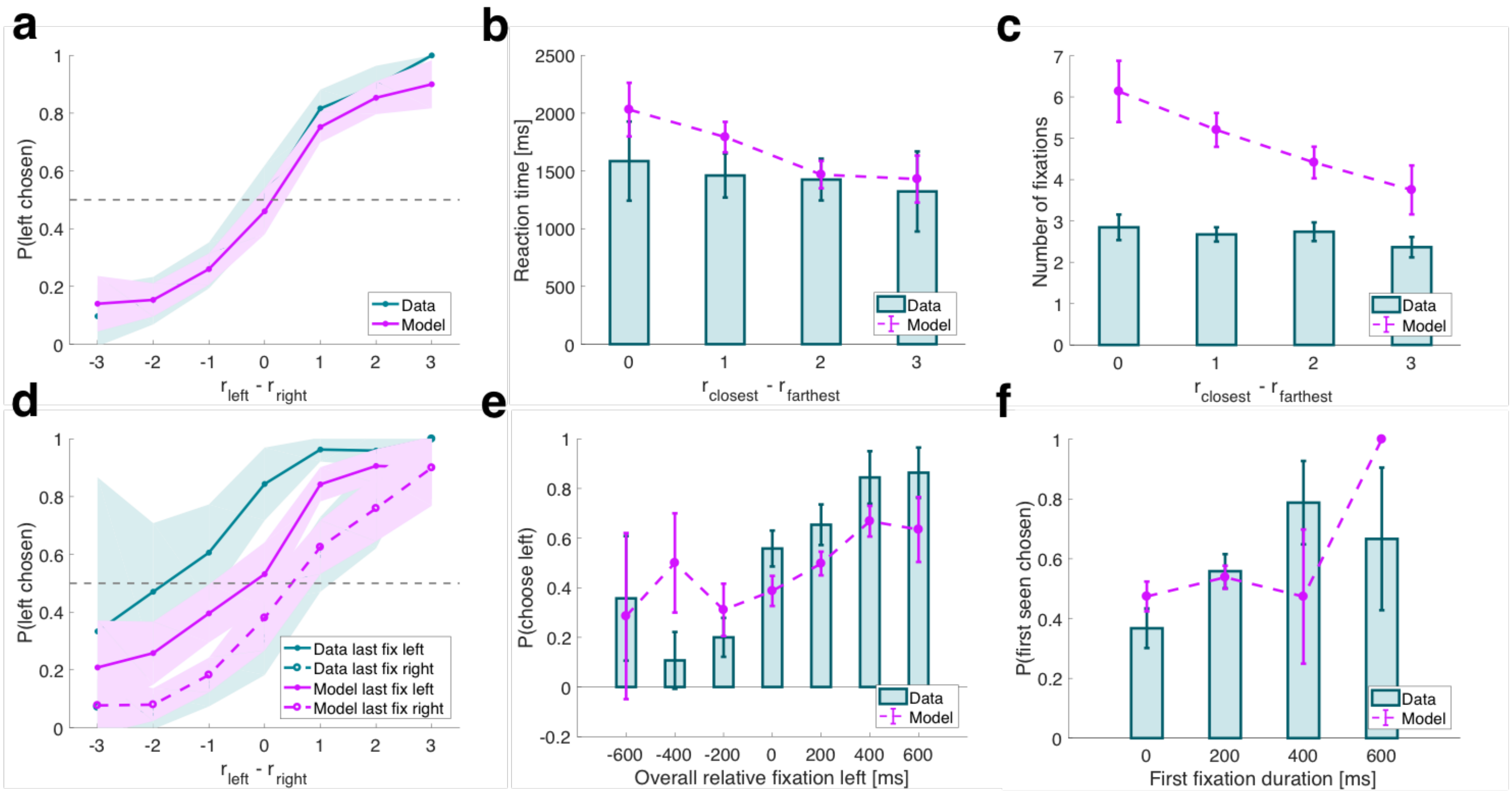

Figure S23. Results for subject 17. Fitted aDDM parameters: $d = 0.0099, \sigma = 0.074, \theta = 0.27$. (**a**) Psychometric choice curve. (**b**) RT curve depicting mean response times versus trial ease, as measured by the difference in absolute proximity between the correct and incorrect options. (**c**) Mean number of fixations versus trial ease. (**d**) Psychometric choice curves conditioned on the location of the last fixation. (**e**) Probability that left item is chosen as a function of the excess amount of time for which the left item was fixated during the trial. (**f**) Probability that the first-seen item is chosen as a function of the duration of that first fixation. Subject data includes only even-numbered trials. Error bars show 95% confidence intervals for the subject's data pooled across all even trials, and across all simulated trials in the case of the model.

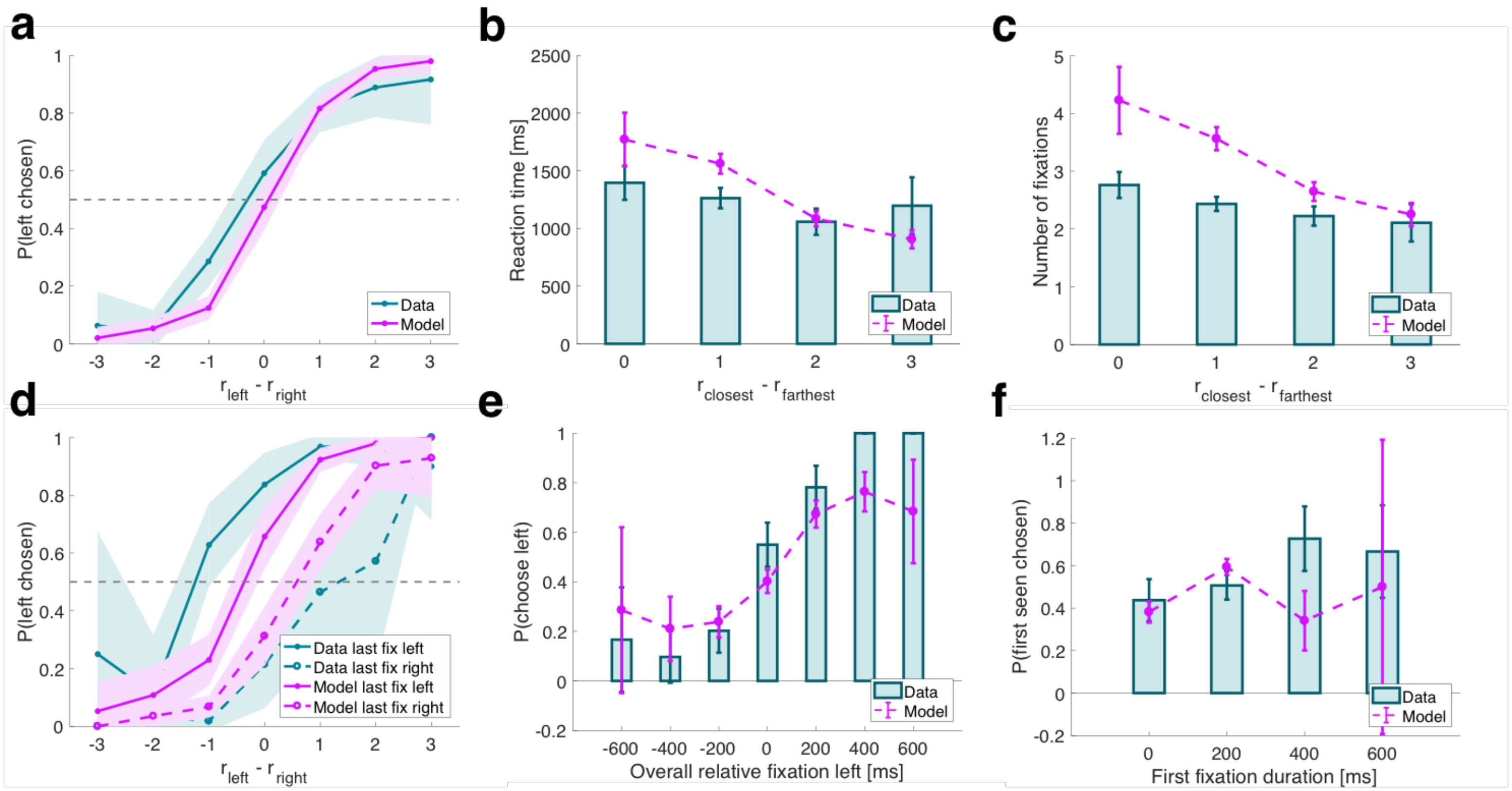

Figure S24. Results for subject 18. Fitted aDDM parameters: $d = 0.0033, \sigma = 0.05, \theta = 0.28$. (**a**) Psychometric choice curve. (**b**) RT curve depicting mean response times versus trial ease, as measured by the difference in absolute proximity between the correct and incorrect options. (**c**) Mean number of fixations versus trial ease. (**d**) Psychometric choice curves conditioned on the location of the last fixation. (**e**) Probability that left item is chosen as a function of the excess amount of time for which the left item was fixated during the trial. (**f**) Probability that the first-seen item is chosen as a function of the duration of that first fixation. Subject data includes only even-numbered trials. Error bars show 95% confidence intervals for the subject's data pooled across all even trials, and across all simulated trials in the case of the model.

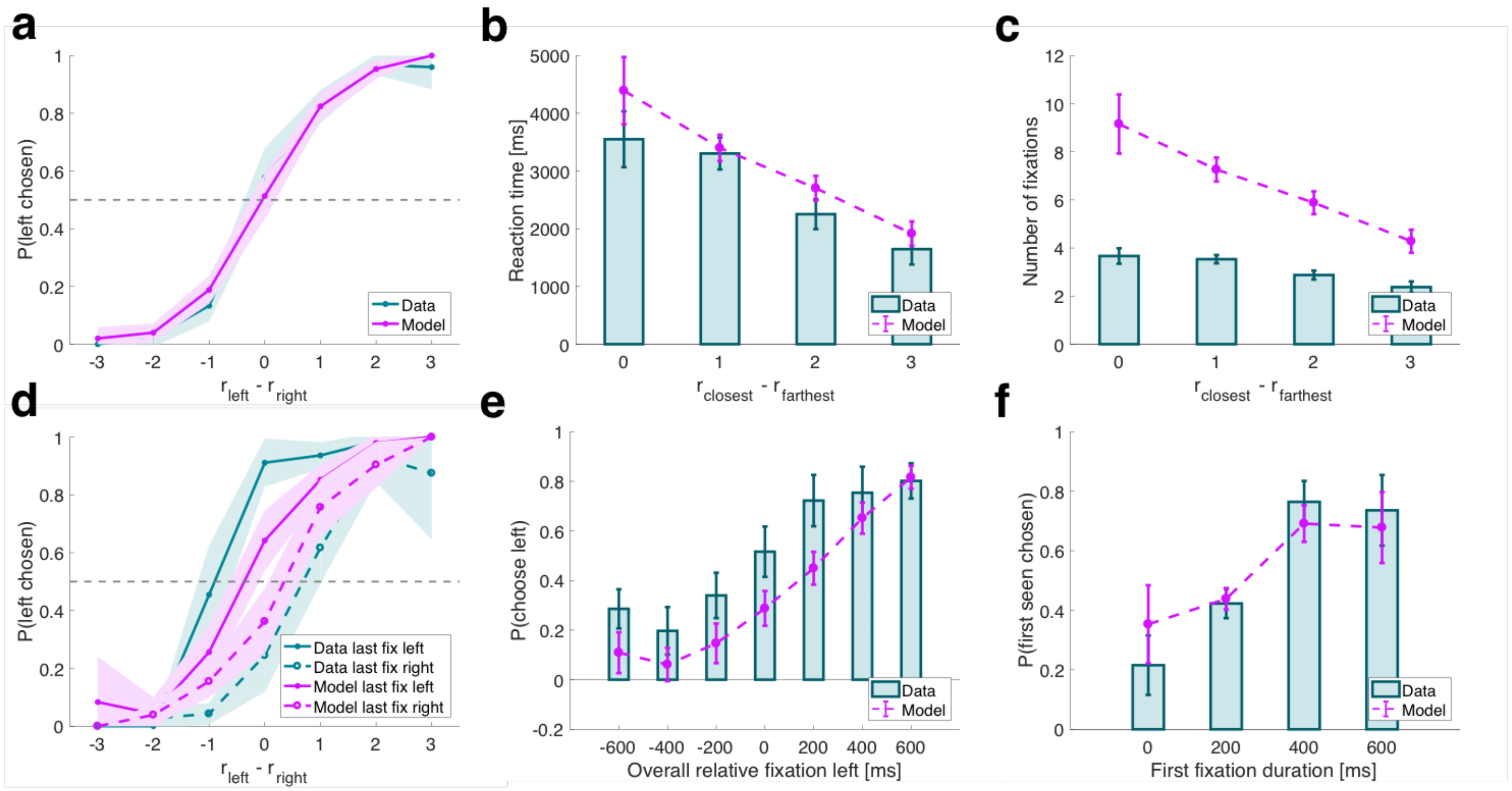

Figure S25. Results for subject 19. Fitted aDDM parameters: $d = 0.006, \sigma = 0.068, \theta = 0.32$. (**a**) Psychometric choice curve. (**b**) RT curve depicting mean response times versus trial ease, as measured by the difference in absolute proximity between the correct and incorrect options. (**c**) Mean number of fixations versus trial ease. (**d**) Psychometric choice curves conditioned on the location of the last fixation. (**e**) Probability that left item is chosen as a function of the excess amount of time for which the left item was fixated during the trial. (**f**) Probability that the first-seen item is chosen as a function of the duration of that first fixation. Subject data includes only even-numbered trials. Error bars show 95% confidence intervals for the subject's data pooled across all even trials, and across all simulated trials in the case of the model.

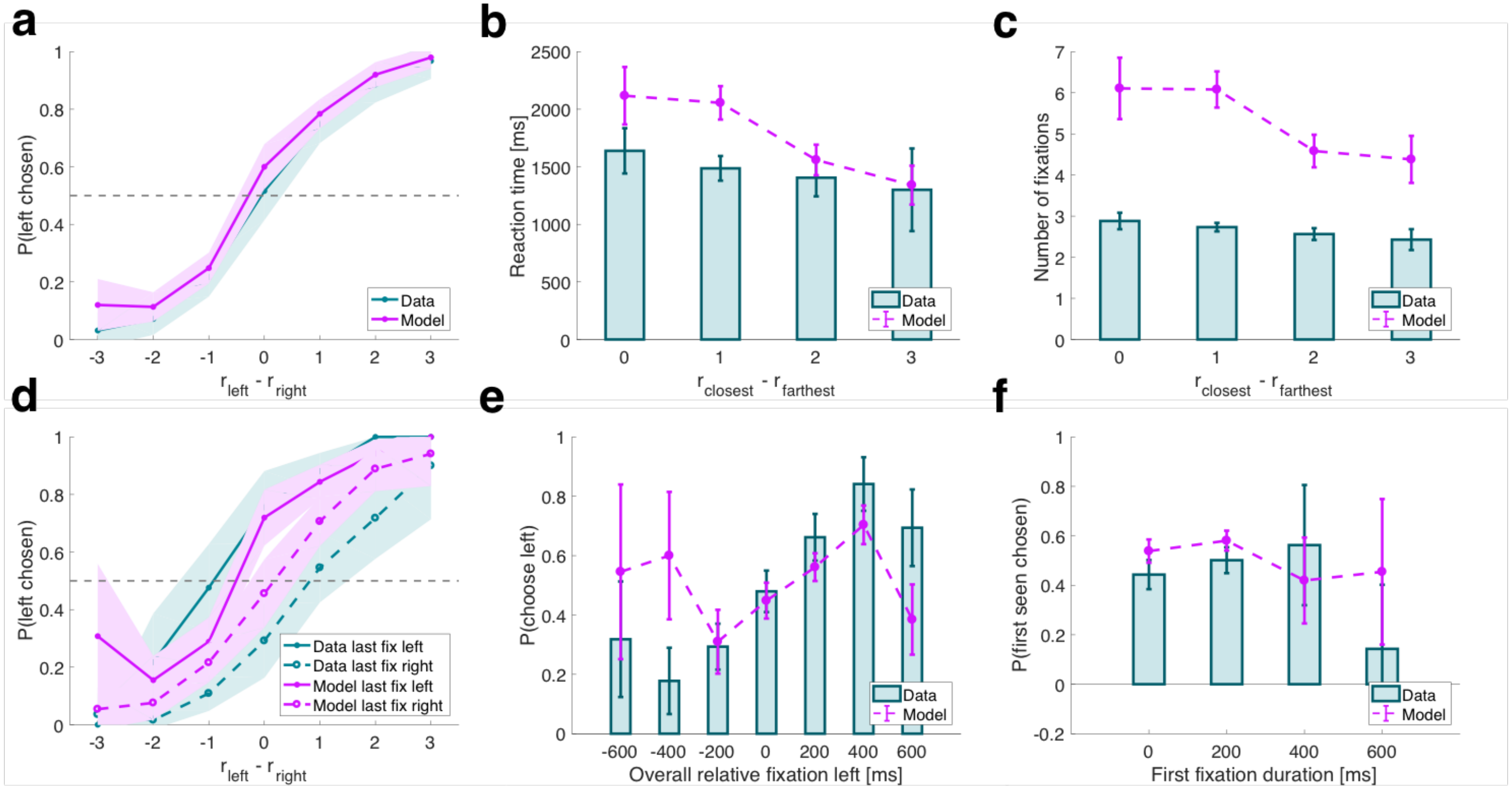

Figure S26. Results for subject 20. Fitted aDDM parameters: $d = 0.0018, \sigma = 0.068, \theta = 0.77$. (**a**) Psychometric choice curve. (**b**) RT curve depicting mean response times versus trial ease, as measured by the difference in absolute proximity between the correct and incorrect options. (**c**) Mean number of fixations versus trial ease. (**d**) Psychometric choice curves conditioned on the location of the last fixation. (**e**) Probability that left item is chosen as a function of the excess amount of time for which the left item was fixated during the trial. (**f**) Probability that the first-seen item is chosen as a function of the duration of that first fixation. Subject data includes only even-numbered trials. Error bars show 95% confidence intervals for the subject's data pooled across all even trials, and across all simulated trials in the case of the model.

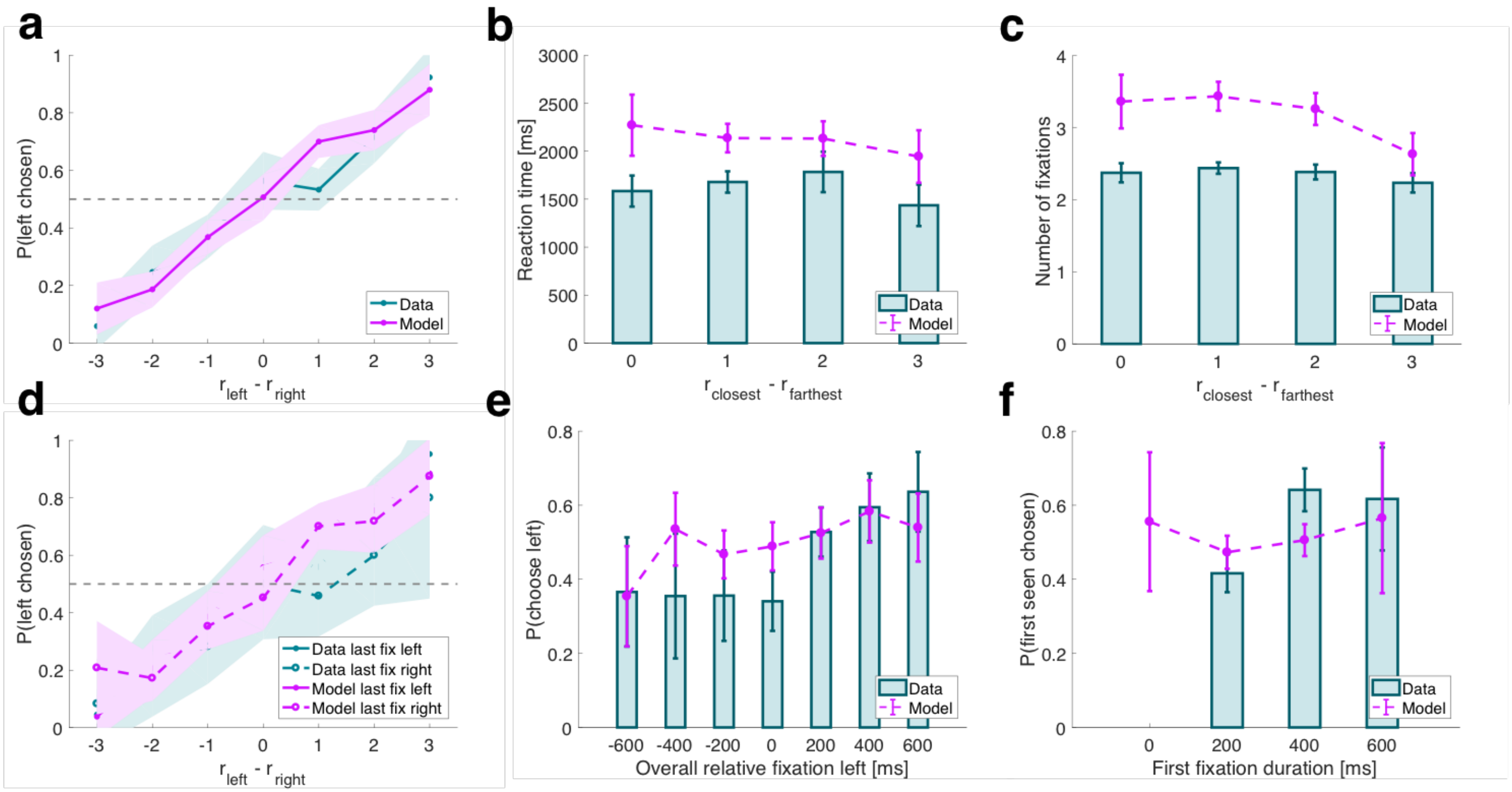

Figure S27. Results for subject 21. Fitted aDDM parameters: $d = 0.0034, \sigma = 0.055, \theta = 0.37$. (**a**) Psychometric choice curve. (**b**) RT curve depicting mean response times versus trial ease, as measured by the difference in absolute proximity between the correct and incorrect options. (**c**) Mean number of fixations versus trial ease. (**d**) Psychometric choice curves conditioned on the location of the last fixation. (**e**) Probability that left item is chosen as a function of the excess amount of time for which the left item was fixated during the trial. (**f**) Probability that the first-seen item is chosen as a function of the duration of that first fixation. Subject data includes only even-numbered trials. Error bars show 95% confidence intervals for the subject's data pooled across all even trials, and across all simulated trials in the case of the model.

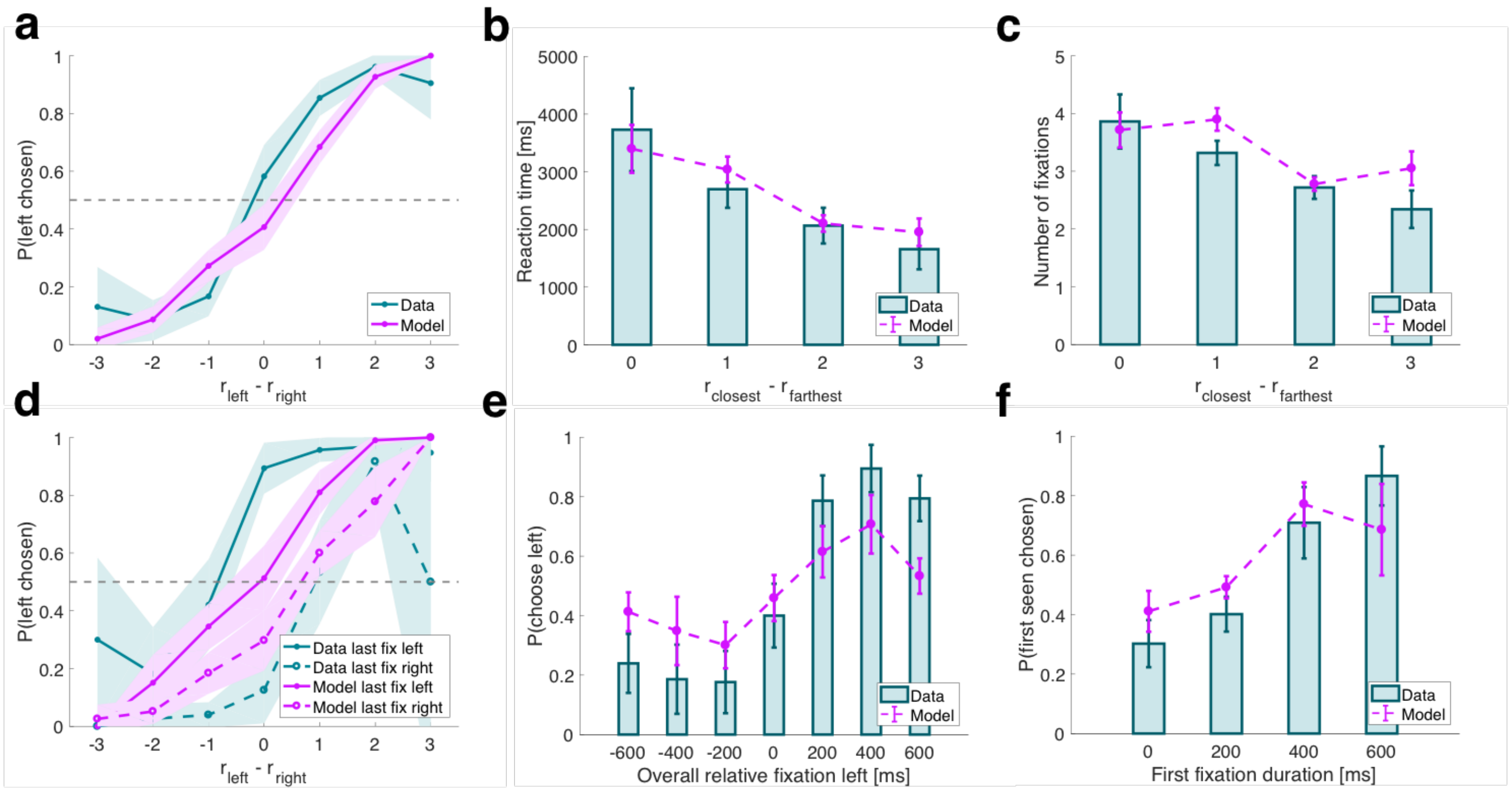

Figure S28. Results for subject 22. Fitted aDDM parameters: $d = 0.0023, \sigma = 0.053, \theta = 0.43$. (**a**) Psychometric choice curve. (**b**) RT curve depicting mean response times versus trial ease, as measured by the difference in absolute proximity between the correct and incorrect options. (**c**) Mean number of fixations versus trial ease. (**d**) Psychometric choice curves conditioned on the location of the last fixation. (**e**) Probability that left item is chosen as a function of the excess amount of time for which the left item was fixated during the trial. (**f**) Probability that the first-seen item is chosen as a function of the duration of that first fixation. Subject data includes only even-numbered trials. Error bars show 95% confidence intervals for the subject's data pooled across all even trials, and across all simulated trials in the case of the model.

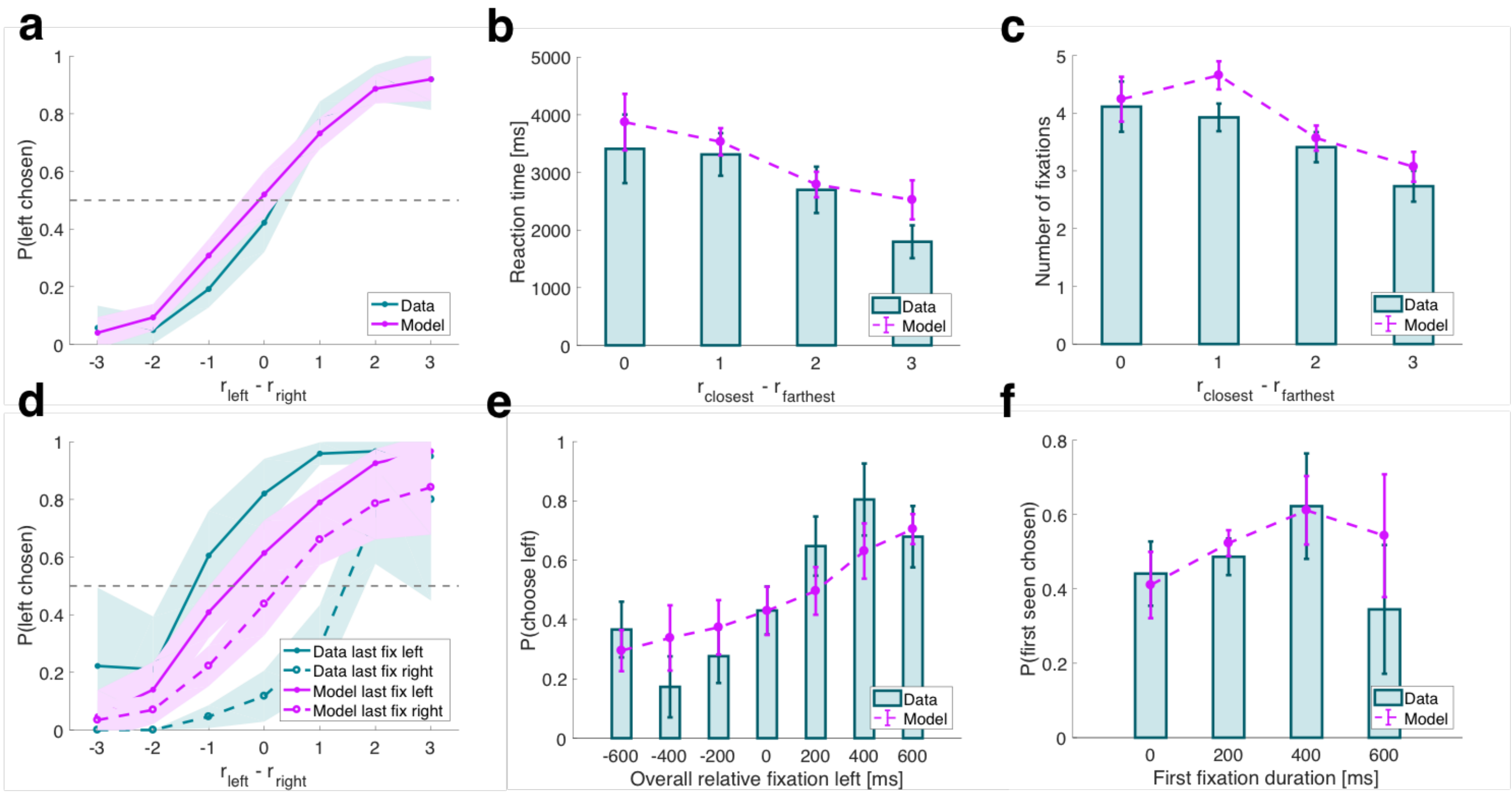

Figure S29. Results for subject 23. Fitted aDDM parameters: $d = 0.0089, \sigma = 0.071, \theta = 0$. (**a**) Psychometric choice curve. (**b**) RT curve depicting mean response times versus trial ease, as measured by the difference in absolute proximity between the correct and incorrect options. (**c**) Mean number of fixations versus trial ease. (**d**) Psychometric choice curves conditioned on the location of the last fixation. (**e**) Probability that left item is chosen as a function of the excess amount of time for which the left item was fixated during the trial. (**f**) Probability that the first-seen item is chosen as a function of the duration of that first fixation. Subject data includes only even-numbered trials. Error bars show 95% confidence intervals for the subject's data pooled across all even trials, and across all simulated trials in the case of the model.

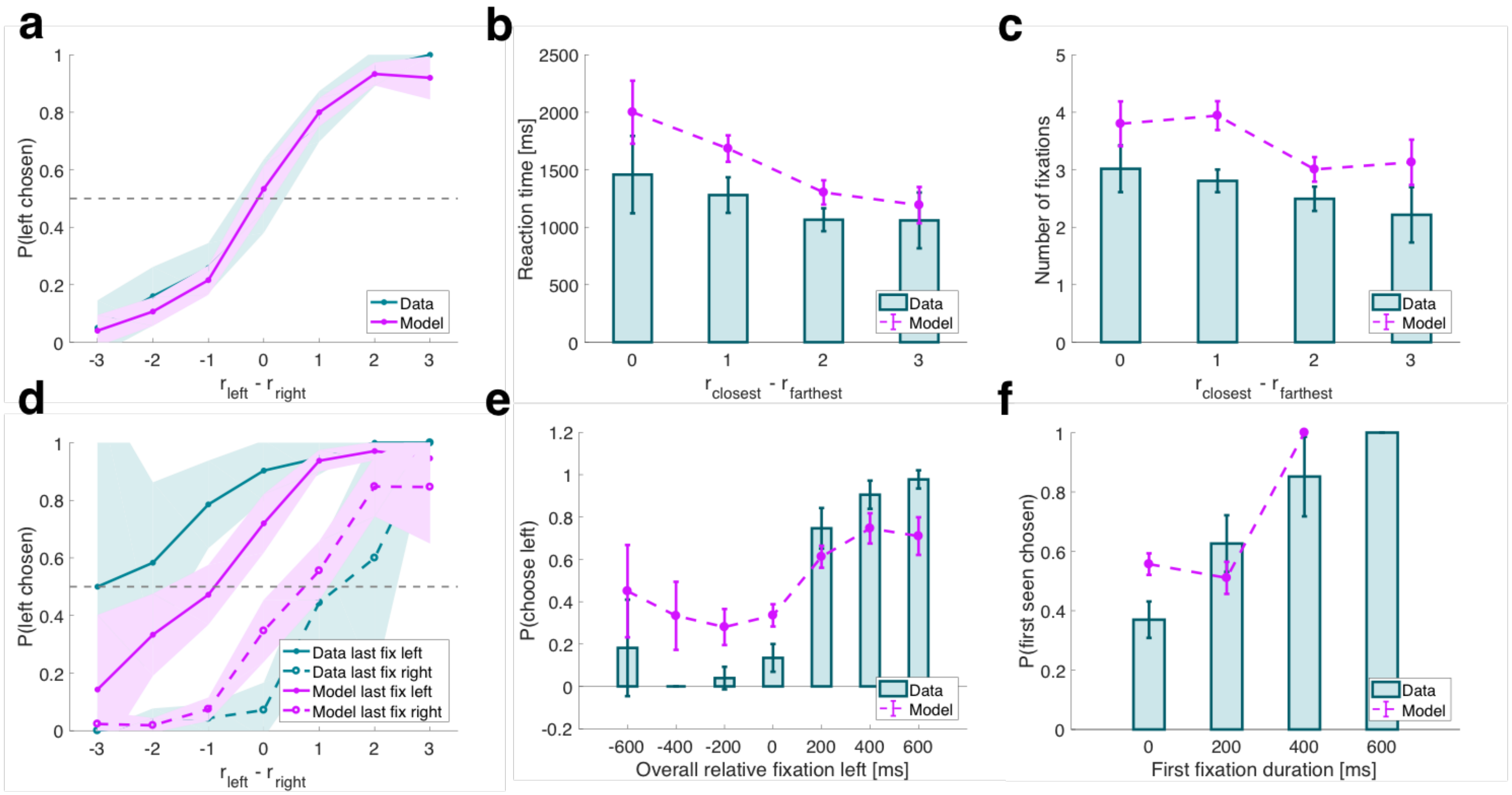

Figure S30. Results for subject 24. Fitted aDDM parameters: $d = 0.0031, \sigma = 0.052, \theta = 0.09$. (**a**) Psychometric choice curve. (**b**) RT curve depicting mean response times versus trial ease, as measured by the difference in absolute proximity between the correct and incorrect options. (**c**) Mean number of fixations versus trial ease. (**d**) Psychometric choice curves conditioned on the location of the last fixation. (**e**) Probability that left item is chosen as a function of the excess amount of time for which the left item was fixated during the trial. (**f**) Probability that the first-seen item is chosen as a function of the duration of that first fixation. Subject data includes only even-numbered trials. Error bars show 95% confidence intervals for the subject's data pooled across all even trials, and across all simulated trials in the case of the model.

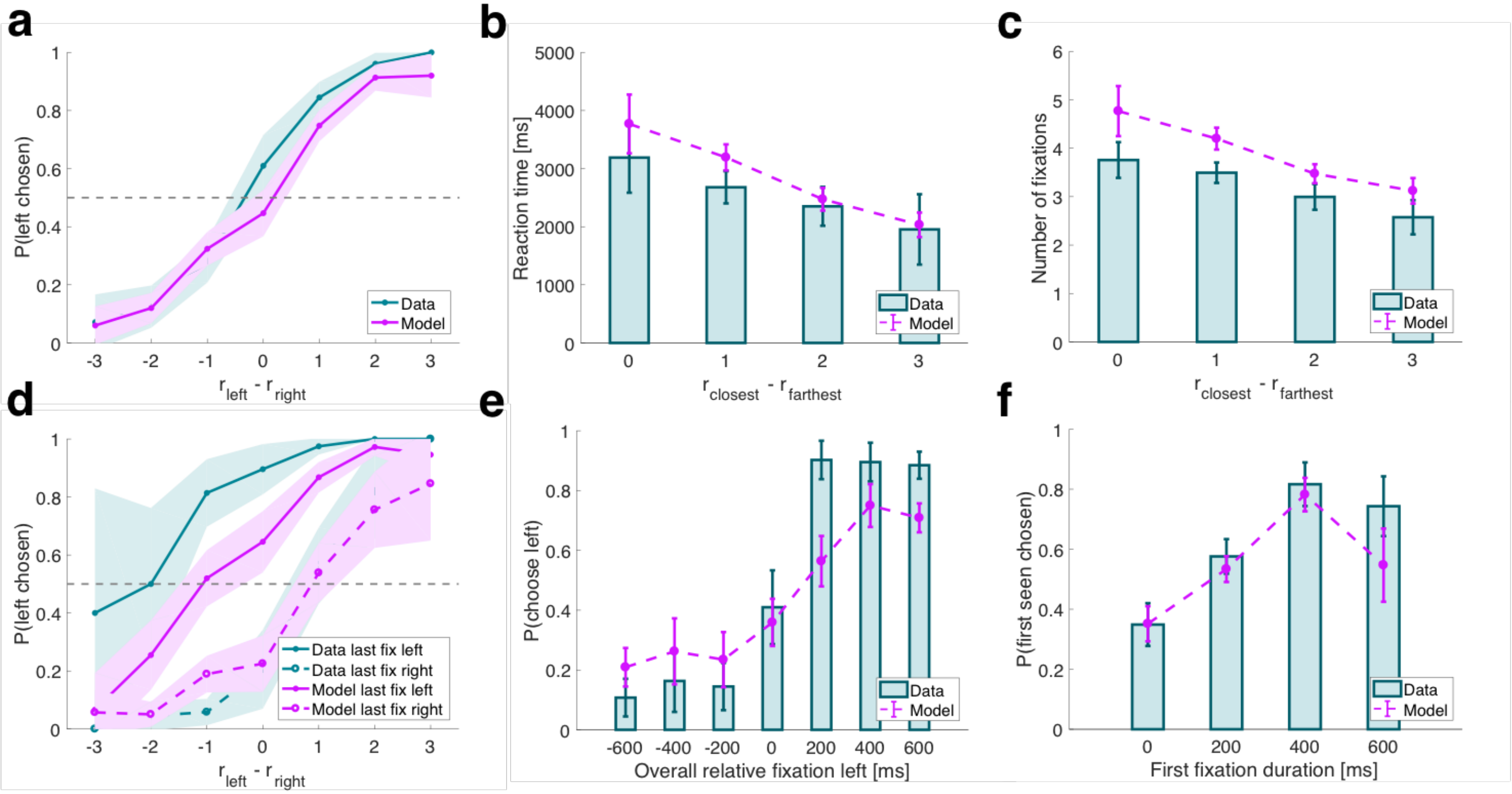

Figure S31. Results for subject 25. Fitted aDDM parameters: $d = 0.0048, \sigma = 0.073, \theta = 0.45$. (**a**) Psychometric choice curve. (**b**) RT curve depicting mean response times versus trial ease, as measured by the difference in absolute proximity between the correct and incorrect options. (**c**) Mean number of fixations versus trial ease. (**d**) Psychometric choice curves conditioned on the location of the last fixation. (**e**) Probability that left item is chosen as a function of the excess amount of time for which the left item was fixated during the trial. (**f**) Probability that the first-seen item is chosen as a function of the duration of that first fixation. Subject data includes only even-numbered trials. Error bars show 95% confidence intervals for the subject's data pooled across all even trials, and across all simulated trials in the case of the model.

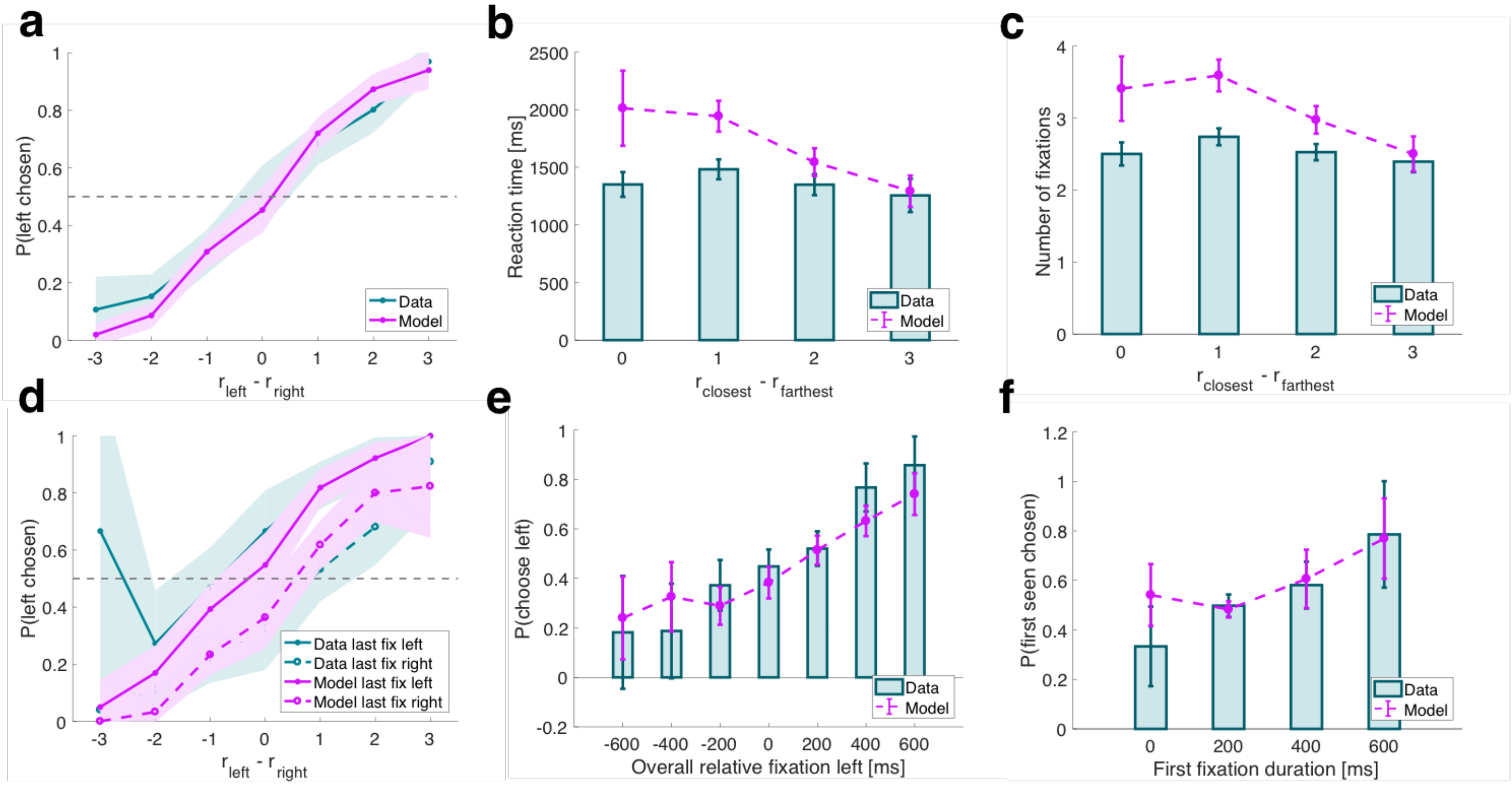

Supplement: Supplementary file 1 [file DataSheet1.pdf]
